# Supplementary material for: Comprehensive dissection of rectal cancer organoids in responses to chemoradiation
Source: Cell Rep Med. 2025 Oct 3;6(10):102397. doi: 10.1016/j.xcrm.2025.102397 (PMC12629783; doi:10.1016/j.xcrm.2025.102397)
Supplement: Document S1. Figures S1–S7 and Tables S1–S12 [file mmc1.pdf]

Cell Reports Medicine, Volume *6*

Supplemental information

Comprehensive dissection of rectal cancer organoids in responses to chemoradiation

Xiaoya Xu, Tao Lv, Ye Yao, Juefeng Wan, Lijun Shen, Fan Xia, Xiaoxue Gao, Yuanchuang Li, Guoxiang Fu, Yun Deng, Mengxue Pan, Qiang Guo, Xinxin Rao, Peiyuan Tang, Xiaomeng Li, Yi Zhou, Liping Liang, Yaqi Wang, Jing Zhang, Hui Zhang, Guichao Li, Min Chen, Junjie Peng, Sanjun Cai, Jianjun Gao, Guoqiang Hua, and Zhen Zhang

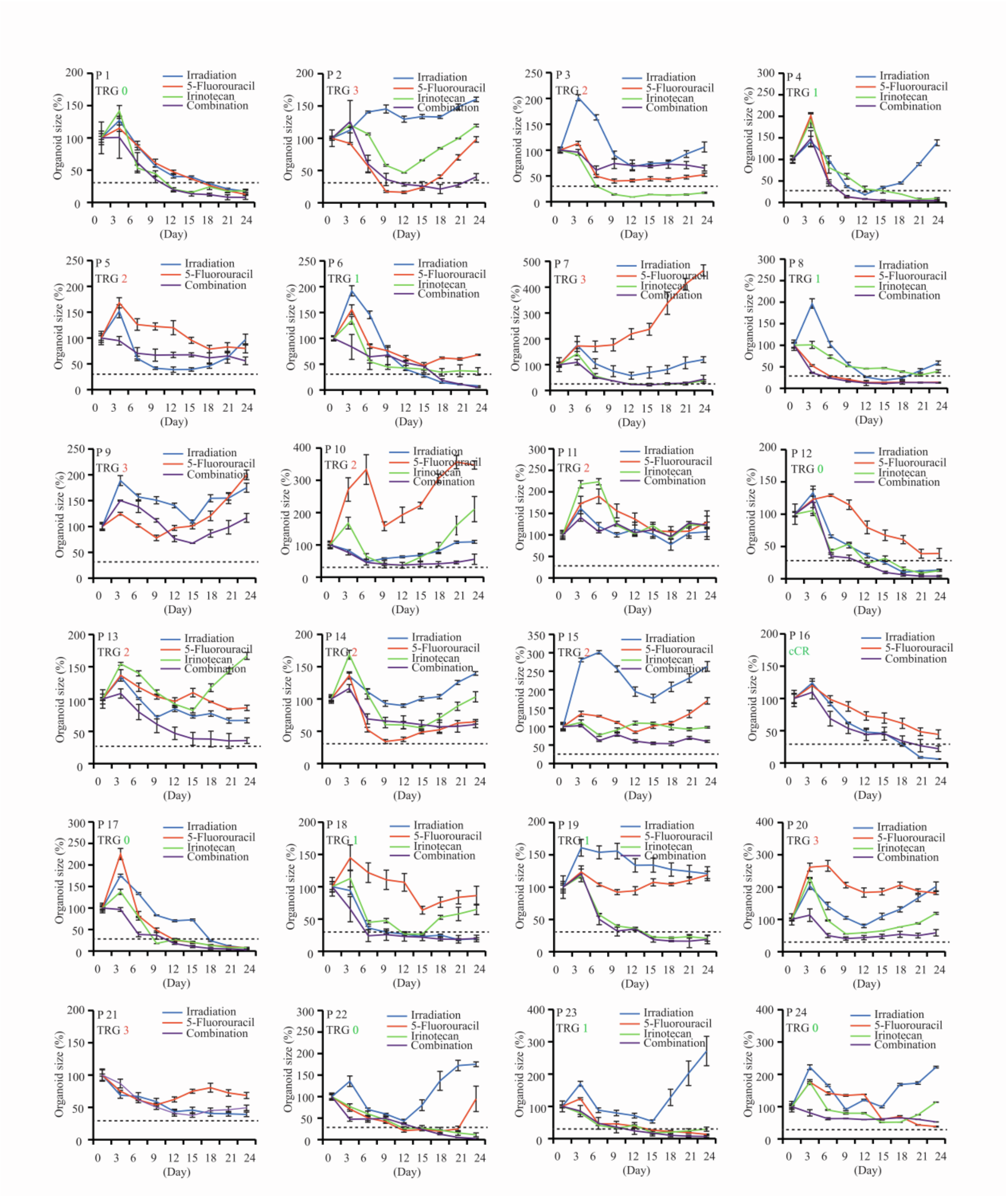

Supplementary Figure 1. The detailed data of RCO lines (P1-P24) size change after single treatments and combined chemoradiation. Organoids size data shown were means from three independent experiments (n = 12). Red curves, patients with a poor clinical response (TRG 2 or 3); green curves, patients with a good clinical response (TRG 0 or 1 or cCR). Dotted lines indicated cutoff of organoid size change (34.87%), Related to Figure 1 and Figure 2.

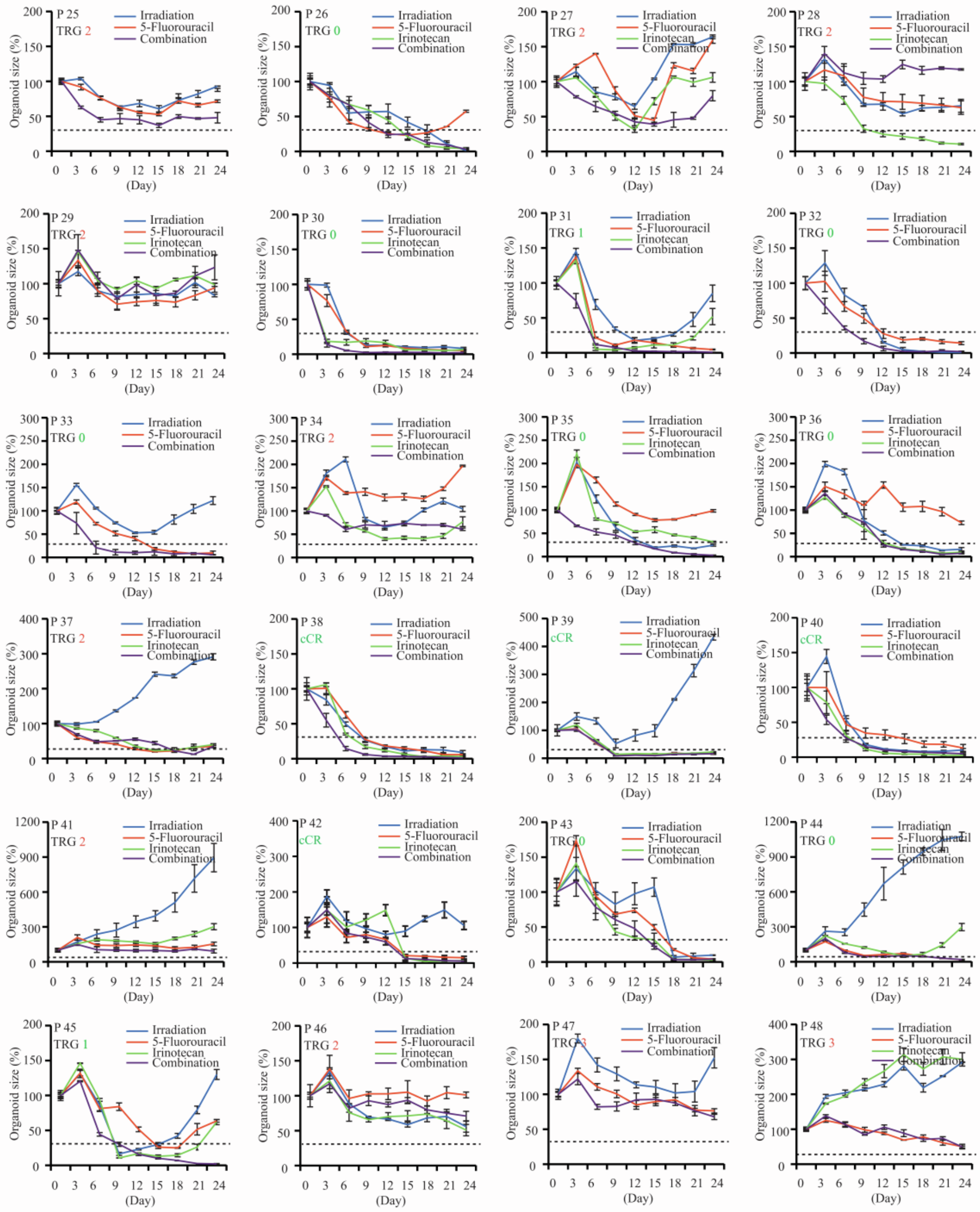

Supplementary Figure 2. The detailed data of RCO lines (P25-P48) size change after single treatments and combined chemoradiation. Organoids size data shown were means from three independent experiments (n = 12). Red curves, patients with a poor clinical response (TRG 2 or 3); green curves, patients with a good clinical response (TRG 0 or 1 or cCR). Dotted lines indicated cutoff of organoid size change (34.87%), Related to Figure 1 and Figure 2.

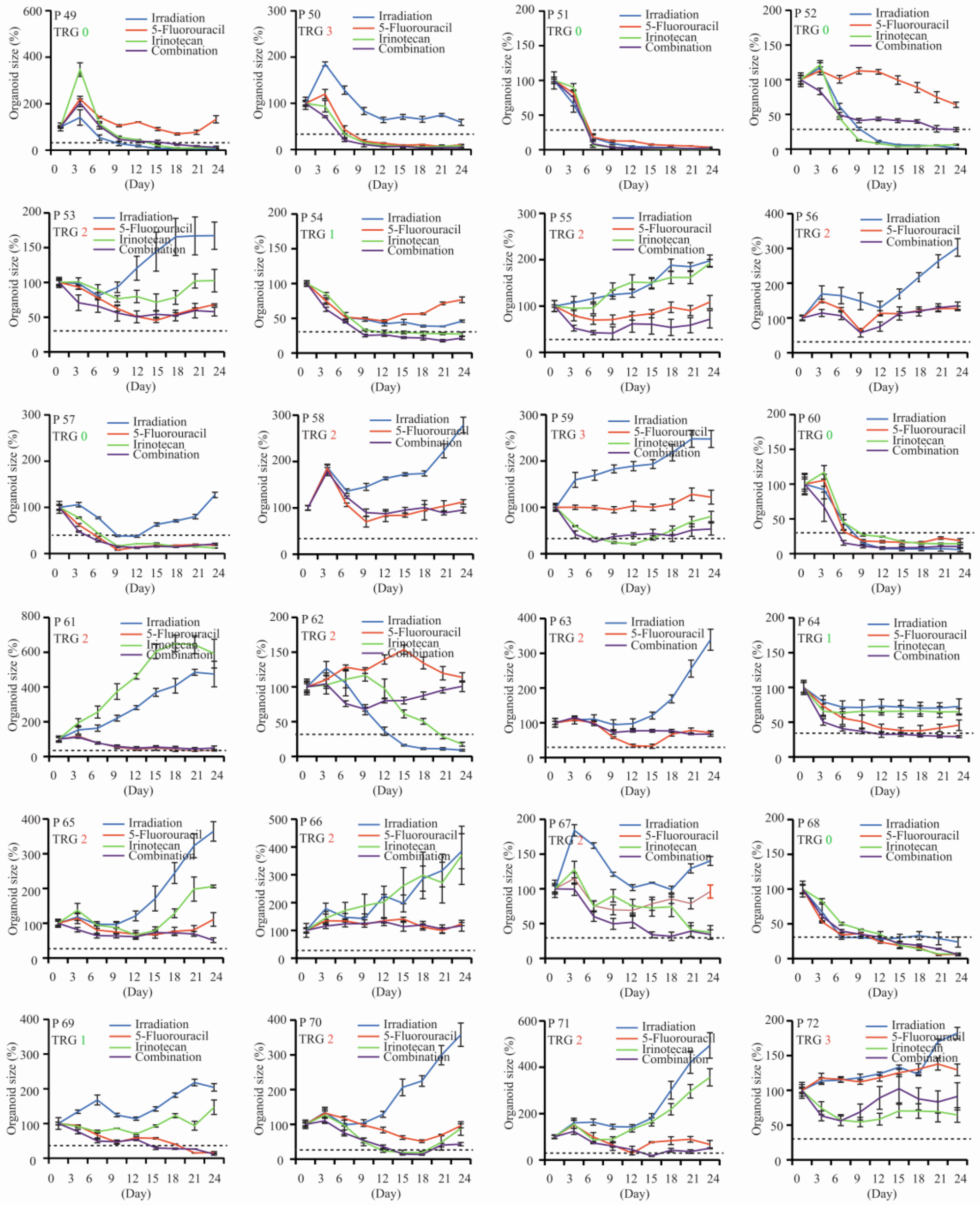

Supplementary Figure 3. The detailed data of RCO lines (P49-P72) size change after single treatments and combined chemoradiation. Organoids size data shown were means from three independent experiments (n = 12). Red curves, patients with a poor clinical response (TRG 2 or 3); green curves, patients with a good clinical response (TRG 0 or 1 or cCR). Dotted lines indicated cutoff of organoid size change (34.87%), Related to Figure 1 and Figure 2.

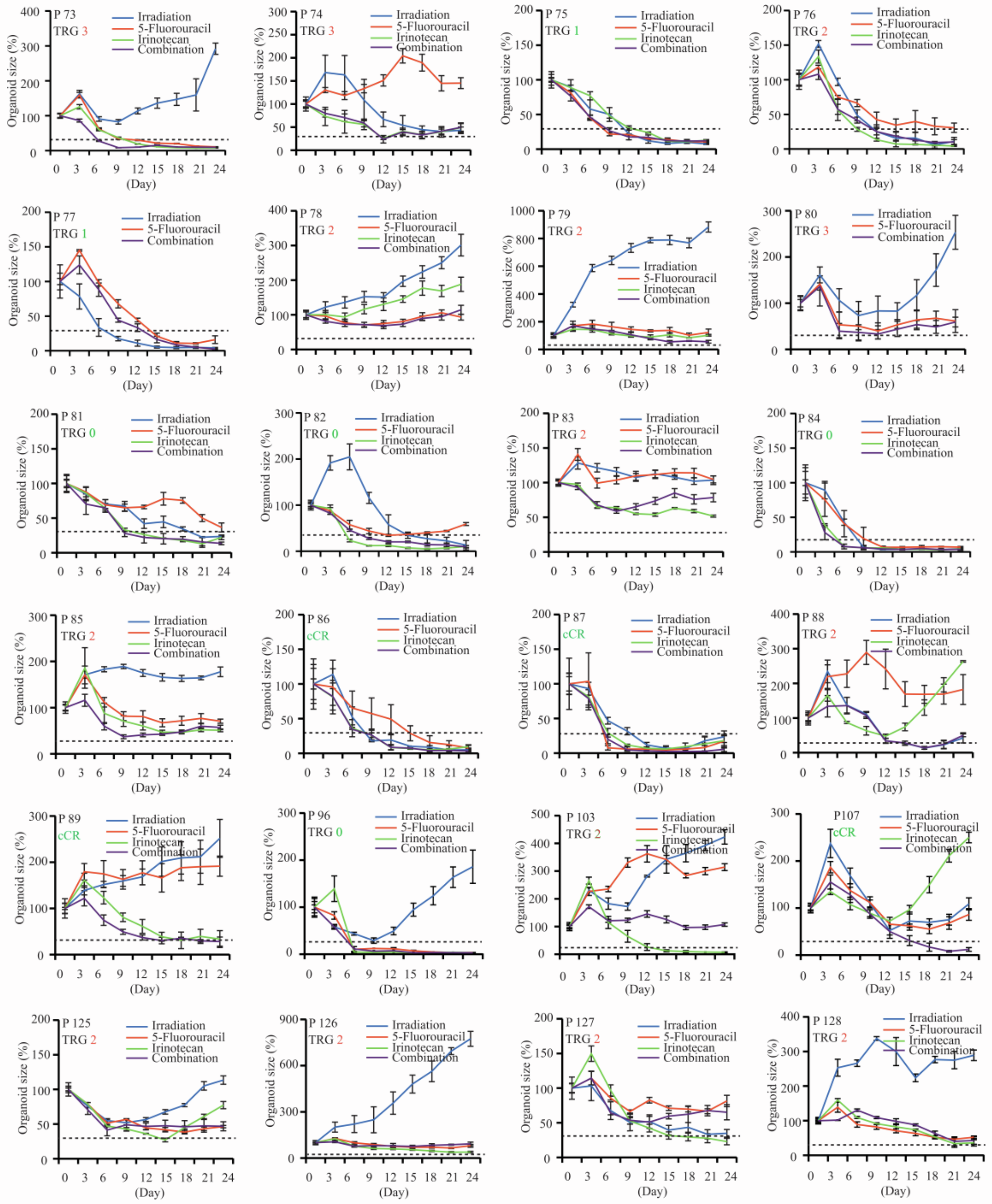

Supplementary Figure 4. The detailed data of RCO lines (P73-P89, P96, P103, P107, P125-P128) size change after single treatments and combined chemoradiation. Organoids size data shown were means from three independent experiments (n = 12). Red curves, patients with a poor clinical response (TRG 2 or 3); green curves, patients with a good clinical response (TRG 0 or 1 or cCR). Dotted lines indicated cutoff of organoid size change (34.87%), Related to Figure 1 and Figure 2.

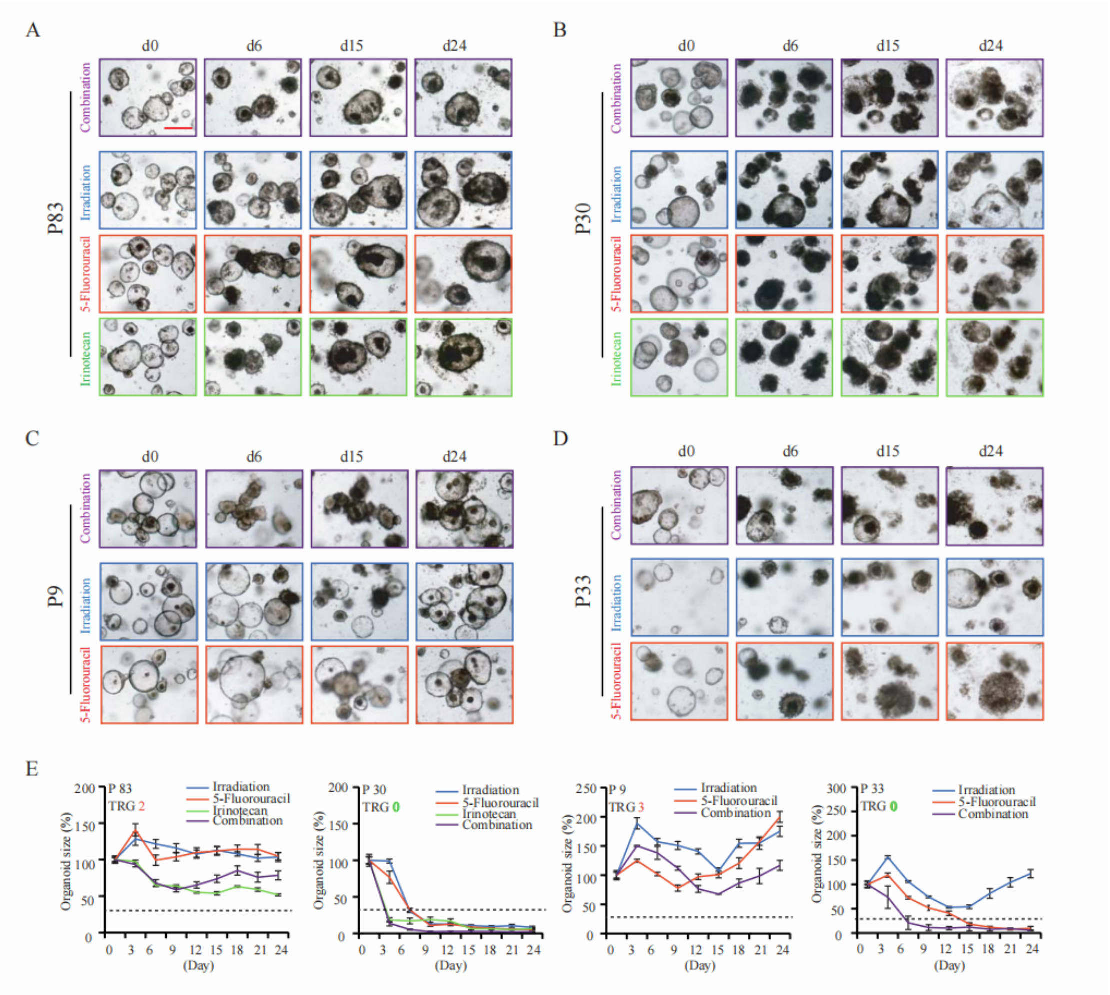

Supplementary Figure 5。Related to Figure 4:

(A,C) Representative bright-filed images of two selected organoid lines (O83 and O9) which were resistant to single treatments of irradiation, 5-Fu and CPT-11 and combined chemoradiation at day0, 6, 15 and 24 after treatments. Scale bars, 200 µm. (B,D) Representative bright-filed images of two selected organoid lines (O30 and O33) which were sensitive to single treatment and combined chemoradiation at day0, 6, 15 and 24 after treatments. (E) Size change curves of organoids after single treatments and combined chemoradiation in 4 selected cases (O83, O30, O9, O33). Organoids size data shown were means±SEM from three independent experiments (n = 12). Red curves, patients with a poor clinical response (TRG 2 or 3); green curves, patients with a good clinical response (TRG 0 or 1 or cCR). Dotted lines indicated cutoff of organoid size change (34.87%).

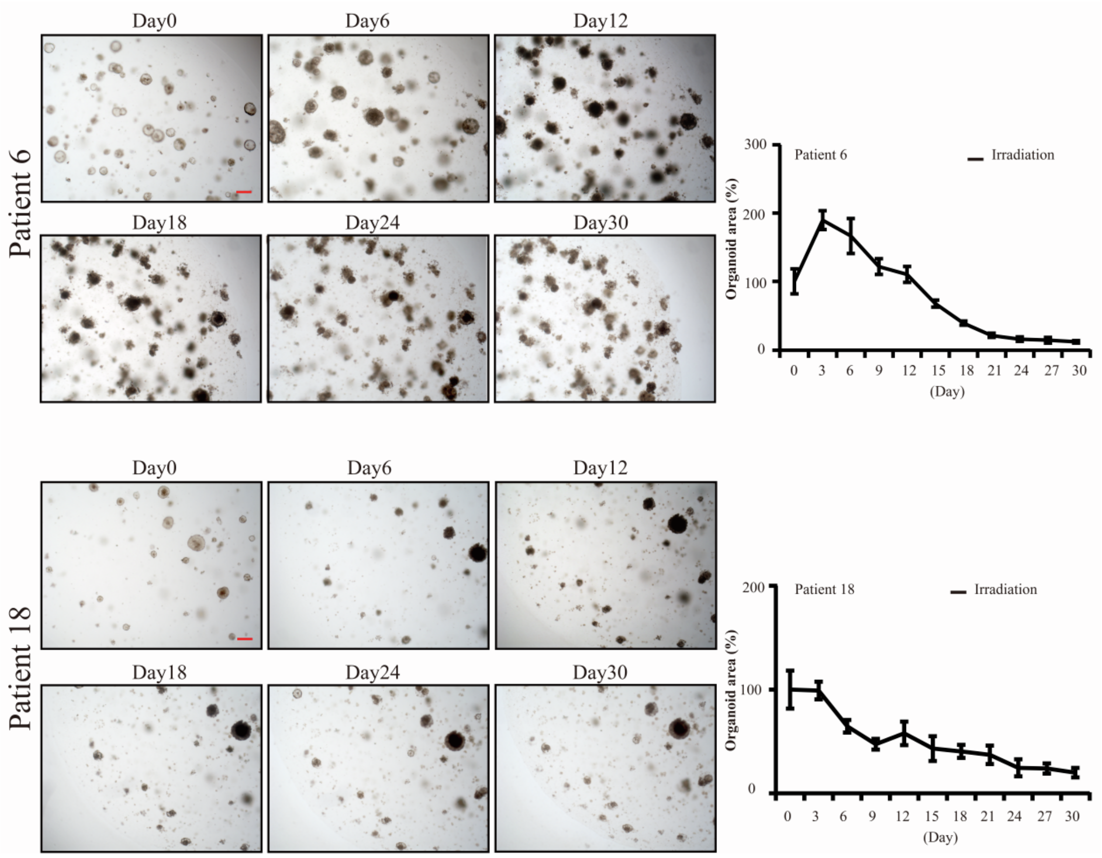

Supplementary Figure 6。 Related to Figure 3.

Representative bright-field images and size change curves of organoids after irradiation in 2 selected cases from day 0 to day 30. Viable organoids exhibited intact and complete structures, whereas non-viable organoids displayed disrupted and fragmented morphology. Organoids size data shown were means±SEM from three independent experiments (n = 12). Scale bar, 200 μm.

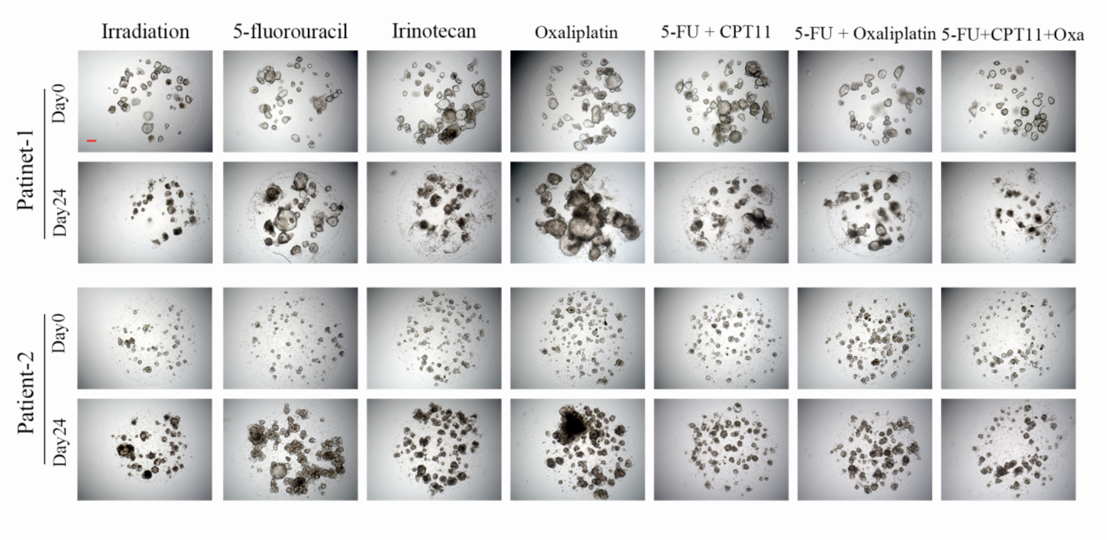

Supplementary Figure 7. Related to Figure 1 and Figure 4.

Representative bright-field images of two RCO lines at day 0 and day 24 after treatment with single or combined regimens, including FOLFOX, FOLFIRI, and FOLFOXIRI. Viable organoids exhibited intact and complete structures, whereas non-viable organoids displayed disrupted and fragmented morphology. Scale bar, 200 μm.

Supplementary Table 1. Clinical data of 128 patients, Related to Figure 1 and Figure2.

Number	Gender	Age	T	N	Histopathology	Treatments	TRG
Patient-1	male	54	T3	N2b	Adenocarcinoma	radiation/irinotecan/Capecitabine	0
Patient-2	female	34	T3b	N2b	Adenocarcinoma	radiation/irinotecan/Capecitabine	3
Patient-3	female	62	T3b	N2b	Adenocarcinoma	radiation/irinotecan/Capecitabine	2
Patient-4	male	50	T3a	N1c	Adenocarcinoma	radiation/irinotecan/Capecitabine	1
Patient-5	male	49	T4a	N2b	Adenocarcinoma	radiation/Capecitabine	2
Patient-6	female	56	T3a	N1b	Adenocarcinoma	radiation/irinotecan/Capecitabine	1
Patient-7	male	30	T3b	N2b	Adenocarcinoma	radiation/irinotecan/Capecitabine	3
Patient-8	female	25	T3a	N1b	Adenocarcinoma	radiation/irinotecan/Capecitabine	1
Patient-9	male	33	T3b	N2	Adenocarcinoma	radiation/Capecitabine	3
Patient-10	male	49	T3a	N2a	Adenocarcinoma	radiation/irinotecan/Capecitabine	2
Patient-11	female	69	T3c	N2b	Adenocarcinoma	radiation/irinotecan/Capecitabine	2
Patient-12	male	46	T3a	N1b	Adenocarcinoma	radiation/irinotecan/Capecitabine	0
Patient-13	male	55	T3b	N2a	Adenocarcinoma	radiation/irinotecan/Capecitabine	2
Patient-14	male	53	T3a	N2a	Adenocarcinoma	radiation/irinotecan/Capecitabine	2
Patient-15	male	64	T4b	N2a	Adenocarcinoma	radiation/irinotecan/Capecitabine	2
Patient-16	male	52	T3a	N2b	Adenocarcinoma	radiation/Capecitabine	ccr
Patient-17	male	55	T3a	N2a	Adenocarcinoma	radiation/irinotecan/Capecitabine	0
Patient-18	male	61	T3a	N1a	Adenocarcinoma	radiation/irinotecan/Capecitabine	1
Patient-19	female	38	T4b	N2b	Adenocarcinoma	radiation/irinotecan/Capecitabine	1
Patient-20	female	43	T3b	N2b	Adenocarcinoma	radiation/irinotecan/Capecitabine	3
Patient-21	male	64	T3b	N1b	Adenocarcinoma	radiation/Capecitabine	3
Patient-22	male	56	T3	N2b	Adenocarcinoma	radiation/Capecitabine	0
Patient-23	male	54	T3	N1	Adenocarcinoma	radiation/irinotecan/Capecitabine	1
Patient-24	male	56	T2	N2a	Adenocarcinoma	radiation/Capecitabine	0
Patient-25	male	60	T3a	N1b	Adenocarcinoma	radiation/Capecitabine	2
Patient-26	male	51	T3b	N2a	Adenocarcinoma	radiation/irinotecan/Capecitabine	0
Patient-27	male	71	T3b	N1b	Adenocarcinoma	radiation/irinotecan/Capecitabine	2
Patient-28	male	53	T3c	N2b	Adenocarcinoma	radiation/irinotecan/Capecitabine	2
Patient-29	female	63	T3	N2	Adenocarcinoma	radiation/irinotecan/Capecitabine	2
Patient-30	male	52	T3	N1	Adenocarcinoma	radiation/irinotecan/Capecitabine	0
Patient-31	male	53	T3c	N2b	Adenocarcinoma	radiation/irinotecan/Capecitabine	1
Patient-32	female	38	T4b	N2b	Adenocarcinoma	radiation/Capecitabine	0
Patient-33	male	44	T3b	N2a	Adenocarcinoma	radiation/Capecitabine	0
Patient-34	male	35	T3c	N2a	Adenocarcinoma	radiation/irinotecan/Capecitabine	2
Patient-35	male	69	T3a	N2a	Adenocarcinoma	radiation/irinotecan/Capecitabine	0
Patient-36	male	58	T3b	N1b	Adenocarcinoma	radiation/irinotecan/Capecitabine	0
Patient-37	male	65	T3a	N2a	Adenocarcinoma	radiation/irinotecan/Capecitabine	2
Patient-38	male	53	T3a	N2a	Adenocarcinoma	radiation/irinotecan/Capecitabine	ccr
Patient-39	male	39	T2	N1b	Adenocarcinoma	radiation/irinotecan/Capecitabine	ccr
Patient-40	male	36	T3a	N1b	Adenocarcinoma	radiation/irinotecan/Capecitabine	ccr
Patient-41	male	32	T3	N+	Adenocarcinoma	radiation/irinotecan/Capecitabine	2
Patient-42	male	42	T3a	N1a	Adenocarcinoma	radiation/irinotecan/Capecitabine	ccr

Patient-43	male	57	T3a	N2a	Adenocarcinoma	radiation/irinotecan/Capecitabine	0
Patient-44	male	73	T3b	N2b	Adenocarcinoma	radiation/irinotecan/Capecitabine	0
Patient-45	female	53	T3b	N1b	Adenocarcinoma	radiation/irinotecan/Capecitabine	1
Patient-46	male	58	T4	N2a	Adenocarcinoma	radiation/irinotecan/Capecitabine	2
Patient-47	female	67	T2	N0	Adenocarcinoma	radiation/Capecitabine	3
Patient-48	male	53	T3	N+	Adenocarcinoma	radiation/irinotecan/Capecitabine	3
Patient-49	female	52	T3a	N0	Adenocarcinoma	radiation/irinotecan/Capecitabine	0
Patient-50	female	54	T3b	N2	Adenocarcinoma	radiation/irinotecan/Capecitabine	3
Patient-51	male	57	T3b	N2a	Adenocarcinoma	radiation/irinotecan/Capecitabine	0
Patient-52	male	38	T3a	N1a	Adenocarcinoma	radiation/irinotecan/Capecitabine	0
Patient-53	male	61	T3a	N1b	Adenocarcinoma	radiation/irinotecan/Capecitabine	2
Patient-54	male	58	T3a	N2a	Adenocarcinoma	radiation/irinotecan/Capecitabine	1
Patient-55	male	59	T3b	N1	Adenocarcinoma	radiation/irinotecan/Capecitabine	2
Patient-56	male	41	T3c	N2b	Adenocarcinoma	radiation/Capecitabine	2
Patient-57	male	63	T3	N1	Adenocarcinoma	radiation/irinotecan/Capecitabine	0
Patient-58	male	52	T4a	N2b	Adenocarcinoma	radiation/Capecitabine	2
Patient-59	female	30	T4b	N2a	Adenocarcinoma	radiation/irinotecan/Capecitabine	3
Patient-60	female	31	T2	N0	Adenocarcinoma	radiation/irinotecan/Capecitabine	0
Patient-61	male	43	T4a	N+	Adenocarcinoma	radiation/irinotecan/Capecitabine	2
Patient-62	male	65	T3b	N1b	Adenocarcinoma	radiation/irinotecan/Capecitabine	2
Patient-63	male	60	T3b	N1b	Adenocarcinoma	radiation/Capecitabine	2
Patient-64	female	56	T3b	N2a	Adenocarcinoma	radiation/irinotecan/Capecitabine	1
Patient-65	male	56	T3	N1	Adenocarcinoma	radiation/irinotecan/Capecitabine	2
Patient-66	male	65	T3a	N2a	Adenocarcinoma	radiation/irinotecan/Capecitabine	2
Patient-67	female	58	T3C	N2a	Adenocarcinoma	radiation/irinotecan/Capecitabine	2
Patient-68	female	56	T2	N1a	Adenocarcinoma	radiation/irinotecan/Capecitabine	0
Patient-69	male	56	T3a	N2b	Adenocarcinoma	radiation/irinotecan/Capecitabine	1
Patient-70	male	63	T3a	N2a	Adenocarcinoma	radiation/irinotecan/Capecitabine	2
Patient-71	male	61	T4	N2a	Adenocarcinoma	radiation/irinotecan/Capecitabine	2
Patient-72	male	60	T3	Nx	Adenocarcinoma	radiation/irinotecan/Capecitabine	3
Patient-73	male	64	T3a	N1a	Adenocarcinoma	radiation/irinotecan/Capecitabine	3
Patient-74	male	46	T3b	N1b	Adenocarcinoma	radiation/irinotecan/Capecitabine	2
Patient-75	female	42	T3b	N1b	Adenocarcinoma	radiation/irinotecan/Capecitabine	1
Patient-76	male	44	T2	N1	Adenocarcinoma	radiation/irinotecan/Capecitabine	2
Patient-77	male	51	T3	N1	Adenocarcinoma	radiation/Capecitabine	1
Patient-78	male	47	T3a	N2a	Adenocarcinoma	radiation/irinotecan/Capecitabine	2
Patient-79	female	57	T3	N2	Adenocarcinoma	radiation/irinotecan/Capecitabine	2
Patient-80	male	47	T4a	N2b	Adenocarcinoma	radiation/Capecitabine	3
Patient-81	female	44	T3	N2	Adenocarcinoma	radiation/irinotecan/Capecitabine	0
Patient-82	male	50	T2	N0	Adenocarcinoma	radiation/irinotecan/Capecitabine	0
Patient-83	female	66	T3a	N0	Adenocarcinoma	radiation/Capecitabine	2
Patient-84	male	58	T2	N1b	Adenocarcinoma	radiation/irinotecan/Capecitabine	0
Patient-85	male	54	T3b	N2a	Adenocarcinoma	radiation/irinotecan/Capecitabine	2
Patient-86	male	57	T4	N2	Adenocarcinoma	radiation/Capecitabine	ccr

Patient-87	male	62	T4b	N2b	Adenocarcinoma	radiation/irinotecan/Capecitabine	ccr
Patient-88	female	83	T3a	N2b	Adenocarcinoma	radiation/Capecitabine	2
Patient-89	male	48	T2	N1	Adenocarcinoma	radiation/irinotecan/Capecitabine	ccr
Patient-90	male	64	T3b	N0	Adenocarcinoma	radiation/irinotecan/Capecitabine	2
Patient-91	female	48	T3a	N1b	Adenocarcinoma	radiation/irinotecan/Capecitabine	3
Patient-92	male	72	T2	N0	Adenocarcinoma	radiation/irinotecan/Capecitabine	2
Patient-93	male	72	T3	N+	Adenocarcinoma	radiation/Capecitabine	2
Patient-94	female	59	T3a	N0	Adenocarcinoma	radiation/irinotecan/Capecitabine	3
Patient-95	male	66	T3b	N2	Adenocarcinoma	radiation/irinotecan/Capecitabine	ccr
Patient-96	male	55	T3b	N1a	Adenocarcinoma	radiation/irinotecan/Capecitabine	0
Patient-97	male	55	T3	N3	Adenocarcinoma	radiation/irinotecan/Capecitabine	1
Patient-98	female	66	T4	N2	Adenocarcinoma	radiation/irinotecan/Capecitabine	0
Patient-99	female	33	T3a	N2b	Adenocarcinoma	radiation/irinotecan/Capecitabine	0
Patient-100	female	53	T3a	N0	Adenocarcinoma	radiation/irinotecan/Capecitabine	0
Patient-101	male	68	T3	N1	Adenocarcinoma	radiation/irinotecan/Capecitabine	0
Patient-102	female	69	T2	N0	Adenocarcinoma	radiation/irinotecan/Capecitabine	ccr
Patient-103	male	44	T4b	N1a	Adenocarcinoma	radiation/irinotecan/Capecitabine	2
Patient-104	male	58	T3	N2	Adenocarcinoma	radiation/irinotecan/Capecitabine	2
Patient-105	female	37	T2	N0	Adenocarcinoma	radiation/irinotecan/Capecitabine	ccr
Patient-106	female	65	T4	N2	Adenocarcinoma	radiation/irinotecan/Capecitabine	2
Patient-107	female	49	T2	N1a	Adenocarcinoma	radiation/irinotecan/Capecitabine	ccr
Patient-108	female	56	T3	N0	Adenocarcinoma	radiation/irinotecan/Capecitabine	ccr
Patient-109	male	38	T3	N1	Adenocarcinoma	irinotecan/Capecitabine	2
Patient-110	female	64	T3b	N2a	Adenocarcinoma	radiation/irinotecan/Capecitabine	2
Patient-111	female	48	T3a	N2b	Adenocarcinoma	radiation/irinotecan/Capecitabine	2
Patient-112	female	34	T4b	N2a	Adenocarcinoma	radiation/irinotecan/Capecitabine	2
Patient-113	male	61	T3C	N2a	Adenocarcinoma	radiation/irinotecan/Capecitabine	2
Patient-114	female	48	T3a	N1b	Adenocarcinoma	radiation/irinotecan/Capecitabine	1
Patient-115	female	55	T3C	N2b	Adenocarcinoma	radiation/Capecitabine	2
Patient-116	male	60	T3a	N1b	Adenocarcinoma	radiation/irinotecan/Capecitabine	2
Patient-117	male	70	T3	N0	Adenocarcinoma	radiation/Capecitabine	2
Patient-118	male	53	T3	N+	Adenocarcinoma	radiation/irinotecan/Capecitabine	3
Patient-119	male	40	T3a	N2a	Adenocarcinoma	radiation/irinotecan/Capecitabine	1
Patient-120	male	55	T3a	N1b	Adenocarcinoma	radiation/Capecitabine	0
Patient-121	male	62	T3a	N1b	Adenocarcinoma	radiation/irinotecan/Capecitabine	2
Patient-122	male	50	T3b	N2a	Adenocarcinoma	radiation/Capecitabine	2
Patient-123	male	65	T3a	N1b	Adenocarcinoma	radiation/irinotecan/Capecitabine	2
Patient-124	female	66	T4b	N1a	Adenocarcinoma	radiation/irinotecan/Capecitabine	0
Patient-125	female	63	T3a	N2a	Adenocarcinoma	radiation/irinotecan/Capecitabine	3
Patient-126	male	66	T2	N+	Adenocarcinoma	radiation/irinotecan/Capecitabine	2
Patient-127	male	49	T3a	N2a	Adenocarcinoma	radiation/irinotecan/Capecitabine	2
Patient-128	male	52	T4a	N2b	Adenocarcinoma	radiation/irinotecan/Capecitabine	2

Supplementary Table 2. Ratios of RCO size change on day24 to day0 after single treatments and combined chemoradiation, Related to Figure 1 and Figure 2.

Patients number	Irradiation	5-fluorouracil 6-(5-Fu)	Irinotecan (CPT-11)	Combined treatments
**Patient1**	0.1732	0.1245	0.1796	0.0779
**Patient2**	1.602	0.9826	1.1959	0.4014
**Patient3**	1.0596	0.5233	0.1737	0.6521
**Patient4**	1.3869	0.0449	0.0947	0.0426
**Patient5**	0.9694	0.7991	0.7702	0.5579
**Patient6**	0.082	0.6799	0.3706	0.0892
**Patient7**	1.1961	4.6461	0.3839	0.4358
**Patient8**	0.5862	0.1408	0.3961	0.1292
**Patient9**	1.7505	1.9983	1.7603	1.1668
**Patient10**	1.0941	3.4739	2.1068	0.55627
**Patient11**	1.0694	1.2971	1.2187	1.2265
**Patient12**	0.1331	0.3925	0.1256	0.0406
**Patient13**	0.6687	0.8629	1.6675	0.3573
**Patient14**	1.3953	0.646	1.0329	0.6052
**Patient15**	2.6321	1.6992	0.9797	0.6007
**Patient16**	0.0615	0.4441	0.0553	0.2211
**Patient17**	0.0674	0.0763	0.0763	0.0301
**Patient18**	0.1946	0.8631	0.6485	0.1989
**Patient19**	1.2079	1.1885	0.2629	0.2374
**Patient20**	2.021	1.8163	1.1856	0.5838
**Patient21**	0.3915	0.6838	0.4642	0.4927
**Patient22**	1.7514	0.9472	0.112	0.0259
**Patient23**	2.7133	0.1318	0.2942	0.0666
**Patient24**	2.223	0.3833	1.1384	0.5263
**Patient25**	0.9302	0.7154	0.8771	0.48
**Patient26**	0.0134	0.5752	0.0419	0.0316
**Patient27**	1.6408	1.5948	1.0612	0.7979
**Patient28**	0.6528	0.6274	0.1055	1.1741
**Patient29**	0.8309	0.9402	0.9843	1.2334
**Patient30**	0.0828	0.0539	0.0663	0.0227
**Patient31**	0.8513	0.0474	0.5182	0.0091
**Patient32**	0.0092	0.1414	0.0248	0.0203
**Patient33**	1.222	0.0974	1.7817	0.0579
**Patient34**	1.0433	1.9676	0.7693	0.6068
**Patient35**	0.247	0.9842	0.3182	0.0291
**Patient36**	0.1601	0.7266	0.0941	0.0733
**Patient37**	2.9111	0.3449	0.3988	0.3476
**Patient38**	0.0891	0.0583	0.0301	0.0111
**Patient39**	4.3328	0.1484	0.2274	0.1635
**Patient40**	3.5075	0.1324	0.0245	0.0486

**Patient41**	8.9479	1.5382	3.0217	0.9244
**Patient42**	1.8199	0.1501	0.0666	0.0541
**Patient43**	0.0964	0.0417	0.0275	0.041
**Patient44**	10.7701	0.1794	2.9778	0.1885
**Patient45**	1.3001	0.6362	0.6196	0.0196
**Patient46**	0.5568	1.0107	0.5072	0.7091
**Patient47**	1.5233	0.7623	0.0781	0.6767
**Patient48**	2.9192	0.5027	3.0001	0.4926
**Patient49**	7.943	1.3261	0.107	0.1197
**Patient50**	3.7136	0.101	0.0824	0.0451
**Patient51**	0.5136	0.0354	0.0139	0.0229
**Patient52**	1.6379	0.6386	0.0624	0.2814
**Patient53**	1.6725	0.6802	1.025	0.5789
**Patient54**	0.4593	0.7663	0.2726	0.2157
**Patient55**	1.9776	1.0877	1.9224	0.7161
**Patient56**	3.029	1.2754	1.2302	1.3519
**Patient57**	4.2972	0.1825	0.1209	0.2077
**Patient58**	2.7879	1.128	0.2522	0.9556
**Patient59**	2.477	1.2204	0.8038	0.5341
**Patient60**	1.1626	0.1873	0.1441	0.1017
**Patient61**	4.7457	0.3593	5.9216	0.4942
**Patient62**	0.0888	1.1369	0.175	1.0093
**Patient63**	3.3909	0.7168	1.8737	0.6753
**Patient64**	0.7278	0.456	0.6503	0.2921
**Patient65**	3.6419	1.1073	2.0594	0.5112
**Patient66**	3.8453	1.229	3.6988	1.1679
**Patient67**	1.401	0.9609	0.3735	0.3429
**Patient68**	0.2383	0.0604	0.0667	0.057
**Patient69**	6.3307	0.1623	1.4695	0.1182
**Patient70**	3.5791	0.9575	0.8799	0.4298
**Patient71**	4.9398	0.6673	3.5636	0.5014
**Patient72**	1.8227	1.2946	0.6457	0.9127
**Patient73**	5.7599	0.1081	0.0762	0.0954
**Patient74**	1.1349	0.4632	0.7727	0.4683
**Patient75**	0.0741	0.1051	0.1271	0.1206
**Patient76**	7.2763	0.3065	0.0427	0.1015
**Patient77**	0.7775	0.16	0.0163	0.024
**Patient78**	3.0112	0.9331	1.8804	1.1438
**Patient79**	8.8329	1.2211	1.0192	0.5536
**Patient80**	2.5331	0.613	0.909	0.5916
**Patient81**	0.2354	0.3641	0.2227	0.1352
**Patient82**	2.2886	0.5901	0.1026	0.0972
**Patient83**	1.0349	1.0459	0.517	0.7846
**Patient84**	9.1641	0.0696	0.0469	0.0373

**Patient85**	1.7749	0.7071	0.5082	0.5723
**Patient86**	0.0487	0.0761	0.0952	0.032
**Patient87**	0.2364	0.1724	0.1803	0.0547
**Patient88**	0.4267	1.8225	2.6342	0.5005
**Patient89**	2.5151	1.914	0.3362	0.2828
**Patient90**	1.2311	0.8607	0.5343	0.7652
**Patient91**	19.8914	2.1416	5.7191	1.0986
**Patient92**	1.3194	0.5599	0.9099	0.7524
**Patient93**	0.3965	2.2952	1.6997	0.6381
**Patient94**	2.5292	1.8019	1.735	1.5187
**Patient95**	1.5039	0.0563	0.619	0.0097
**Patient96**	1.6662	0.0703	0.0222	0.029
**Patient97**	7.8834	3.7457	2.5245	0.0314
**Patient98**	0.043	0.2199	0.0239	0.0135
**Patient99**	0.0602	0.0663	0.0363	0.0105
**Patient100**	0.0792	0.1155	0.0645	0.0308
**Patient101**	1.0608	0.3499	0.3253	0.0576
**Patient102**	0.0111	0.023	0.016	0.0057
**Patient103**	5.0088	3.8364	0.1415	1.0696
**Patient104**	2.8429	0.3234	0.7362	0.3454
**Patient105**	0.602	0.5201	0.5798	0.6035
**Patient106**	1.6517	0.6862	0.7521	0.4515
**Patient107**	0.7926	1.2499	1.8746	0.1785
**Patient108**	4.5186	0.0901	0.0209	0.0304
**Patient109**	0.8927	0.8919	0.8277	0.7425
**Patient110**	1.5705	1.3677	0.626	0.7442
**Patient111**	0.7304	0.7564	0.4828	0.9134
**Patient112**	2.0702	0.781	0.8651	0.8276
**Patient113**	1.2093	1.0782	1.0998	0.7589
**Patient114**	5.5238	0.1418	0.0447	0.0308
**Patient115**	6.1799	2.9776	4.3813	1.1169
**Patient116**	0.7552	0.9322	0.8107	0.6934
**Patient117**	0.9604	0.6514	1.142	0.7548
**Patient118**	2.4642	0.3975	0.6116	0.3857
**Patient119**	2.4711	0.7668	0.0667	0.3415
**Patient120**	0.1434	0.181	0.6209	0.0592
**Patient121**	2.5753	0.8419	1.0003	0.498
**Patient122**	4.2256	3.8528	1.0073	1.7538
**Patient123**	1.9527	0.4455	0.0888	0.6037
**Patient124**	1.7715	0.6291	0.7631	0.4641
**Patient125**	0.4327	1.4518	0.4895	0.4951
**Patient126**	7.7413	0.8003	0.3873	0.9186
**Patient127**	0.3488	0.8148	0.229	0.6516
**Patient128**	2.8906	0.5223	0.3577	0.4272

Supplementary Table 3. Diagnostic test of responses of RCO to combined chemoradiation for predicting the clinical outcomes of LARC patients in the discovery cohort, Related to Figure 2.

**Organoids**	**Clinical Outcomes**		
	good	poor	Total
sensitive	36	5	41
resistant	1	38	39
Total	37	43	80

Accuracy(%)=(36+38)/(41+49)=92.50%

Sensitivity(%)=38/(38+5)=88.37%

Specifity(%)=36/((36+1)=97.30%

Supplementary Table 4. A chi-square test of independence to examine the relation between RCO responses to cominbed chemoradiation and single treatments to predict the clnical outcomes of LARC patientsin the discovery cohort, Related to Figure2.

**Combined chemoradiation**	**Single treatments**		
	sensitive	resistant	Total
sensitive	38	3	41
resistant	3	36	39
Total	41	39	80

Supplementary Table 5. Diagnostic test of responses of RCO to combined chemoradiation for predicting the clinical outcomes of LARC patients in the validation cohort, Related to Figure 2.

**Organoids**	**Clinical Outcomes**		
	good	poor	Total
sensitive	19	1	20
resistant	2	26	28
Total	21	27	48

Accuracy(%)=(19+26)/(20+28)=93.75%

Sensitivity(%)=26/(26+1)=96.30%

Specifity(%)=19/((2+19)=90.48%

Supplementary Table 6. Diagnostic test of responses of RCO to single treatments for predicting the clinical outcomes of LARC patients in the validation cohort, Related to Figure 2.

Organoids	Clinical Outcomes		
	good	poor	Total
sensitive	17	4	21
resistant	4	23	27
Total	21	27	48

Accuracy(%)=(17+23)/(21+27)=83.33%

Sensitivity(%)=23/(23+4)=85.19%

Specifity(%)=17/((4+17)=80.95%

Supplementary Table 7. A chi-square test of independence to examine the relation between RCO responses to cominbed treatments and single treatments to predict the clnical outcomes of LARC patients in the validation cohort, Related to Figure 2.

Combined chemoradiation	Single treatments		
	Matched	Not matched	Total
Matched	18	2	20
Not matched	3	25	28
Total	21	27	48

Supplementary Table 8. Four modes of organoids responses to combined chemoradiation and tumor regression of corresponding clinical patients, Related to Figure 3

Patients number	Four categories	Clinical response
**Patient1**	rapid	0
**Patient2**	recovery	3
**Patient3**	resistant	2
**Patient4**	rapid	1
**Patient5**	resistant	2
**Patient6**	moderate	1
**Patient7**	recovery	3
**Patient8**	rapid	1
**Patient9**	resistant	3
**Patient10**	resistant	2
**Patient11**	resistant	2
**Patient12**	rapid	0
**Patient13**	resistant	2
**Patient14**	resistant	2
**Patient15**	resistant	2
**Patient16**	moderate	ccr
**Patient17**	rapid	0

**Patient18**	rapid	1
**Patient19**	moderate	1
**Patient20**	resistant	3
**Patient21**	resistant	3
**Patient22**	moderate	0
**Patient23**	rapid	1
**Patient24**	resistant	0
**Patient25**	resistant	2
**Patient26**	rapid	0
**Patient27**	resistant	2
**Patient28**	resistant	2
**Patient29**	resistant	2
**Patient30**	rapid	0
**Patient31**	rapid	1
**Patient32**	rapid	0
**Patient33**	rapid	0
**Patient34**	resistant	2
**Patient35**	rapid	0
**Patient36**	rapid	0
**Patient37**	recovery	2
**Patient38**	rapid	ccr
**Patient39**	rapid	ccr
**Patient40**	rapid	ccr
**Patient41**	resistant	2
**Patient42**	moderate	ccr
**Patient43**	moderate	0
**Patient44**	moderate	0
**Patient45**	rapid	1
**Patient46**	resistant	2
**Patient47**	resistant	3
**Patient48**	resistant	3
**Patient49**	moderate	0
**Patient50**	rapid	3
**Patient51**	rapid	0
**Patient52**	moderate	0
**Patient53**	resistant	2
**Patient54**	rapid	1
**Patient55**	resistant	2
**Patient56**	resistant	2
**Patient57**	rapid	0
**Patient58**	resistant	2
**Patient59**	recovery	3
**Patient60**	rapid	0
**Patient61**	resistant	2

**Patient62**	resistant	2
**Patient63**	resistant	2
**Patient64**	rapid	1
**Patient65**	resistant	2
**Patient66**	resistant	2
**Patient67**	resistant	2
**Patient68**	rapid	0
**Patient69**	moderate	1
**Patient70**	recovery	2
**Patient71**	recovery	2
**Patient72**	resistant	3
**Patient73**	rapid	3
**Patient74**	recovery	2
**Patient75**	rapid	1
**Patient76**	rapid	2
**Patient77**	rapid	1
**Patient78**	resistant	2
**Patient79**	resistant	2
**Patient80**	resistant	3
**Patient81**	rapid	0
**Patient82**	rapid	0
**Patient83**	resistant	2
**Patient84**	rapid	0
**Patient85**	resistant	2
**Patient86**	rapid	ccr
**Patient87**	rapid	ccr
**Patient88**	recovery	2
**Patient89**	moderate	ccr
**Patient96**	rapid	0
**Patient103**	resistant	2
**Patient107**	moderate	ccr
**Patient125**	resistant	3
**Patient126**	resistant	2
**Patient127**	resistant	2
**Patient128**	resistant	2

Supplementary Table 9. A chi-square test of independence to examine the relation among the RCO responses (sensitive or resistant) to irradiation, 5-Fu and CPT-11 in 128 organoid lines, Related to Figure 2.

**Treatments**	**Organoids response**		
	Sensitive	Resistant	Total
Radiation	24	104	128
5-Fu	39	89	128

CPT-11	48	66	114

Supplementary Table 10. A fisher's exact test of independence to examine the relation among the RCO responses (only sensitive or not) to irradiation, 5-Fu and CPT-11 in 128 organoid lines, Related to Figure 2.

**Treatments**	**Organoids response**		
	Sensitive (Only)	Others	Total
Radiation	2	126	128
5-Fu	8	120	128
CPT-11	14	100	114

Supplementary Table 11. Organoids size change ratios on day24 to day0 after treatment with single or combined regimens from 10 cases, Related to Figure 2 and Figure 4.

	Irradiation	5Fu	CPT11	Oxaliplatin	5FU + CPT11	5FU + Oxaliplatin	5FU + CPT11 + Oxaliplatin
**Patient-1**	0.227	1.446	0.182	5.162	0.183	1.041	0.086
**Patient-2**	0.699	3.929	0.267	2.057	0.481	1.281	0.385
**Patient-3**	1.393	2.472	1.463	5.415	1.901	1.911	1.256
**Patient-4**	0.569	0.983	0.495	0.805	0.775	0.806	0.776
**Patient-5**	0.173	0.422	1.160	0.068	0.103	0.112	0.099
**Patient-6**	0.892	0.516	0.123	1.219	0.209	0.528	0.339
**Patient-7**	0.553	2.462	0.272	9.933	0.937	2.444	0.887
**Patient-8**	1.194	0.683	2.424	1.268	0.889	0.499	0.799
**Patient-9**	0.238	0.541	0.136	0.313	0.339	0.452	0.142
**Patient-10**	1.107	0.950	0.982	1.061	1.039	0.798	0.675

Supplementary Table 12. Specific details of the organoid culture conditions, Related to STAR METHOD.

Regent name	Company	Cat No.	Stock	Solvent	Final
Advanced DMEM/F12	Gibco	12634-010	---	1×	1×
R-spondin 1	Sino	11083-	50μg/ mL	0.1%BSA/P	500ng/mL
Noggin	Sino	50688-	10μg/ mL	0.1%BSA/P	100ng/mL
R-spondin 1	EnaMab	ERSP1000	50μg/ mL	0.1%BSA/P	500ng/mL
Noggin	EnaMab	ENGG0100	10μg/ mL	0.1%BSA/P	100ng/mL
Wnt3a	EnaMab	EWNT0100	10μg/ mL	0.1%BSA/P	100ng/mL
EGF	Sino	50482-	500μg/ mL	0.1%BSA/P	50ng/mL
HEPES	Gibco	15630080	100×	---	1×
Glutamax	Gibco	35050061	100×	---	1×
Normocin	InvivoGen	ant-nr-1	500×	---	1×
Gentamicin/amphotericin	Gibco	R01510	500×	---	1×
N2	Invitrogen	17502-048	50×	---	1×
B27	Invitrogen	17504-044	100×	---	1×
n-Acetylcysteine	Sigma-	A9165	500mM	ddH2O	1mM
Niacinamide	Sigma-	N0636	1M	ddH2O	10mM
A-83-01	Tocris	2939	5mM	DMSO	500nM
SB202190	Sigma-	S7067	30M	DMSO	3μM
Gastrin	Sigma-	G9145	100uM	0.1%BSA/P	10nM
Prostaglandin E2	Sigma-	P6532	100uM	DMSO	10nM
