# Supplementary material for: Comprehensive dissection of rectal cancer organoids in responses to chemoradiation
Source: Cell Rep Med. 2025 Oct 3;6(10):102397. doi: 10.1016/j.xcrm.2025.102397 (PMC12629783; doi:10.1016/j.xcrm.2025.102397)
Supplement: Document S2. Article plus supplemental information [file mmc2.pdf]

Cell Reports
Medicine

Article

Comprehensive dissection of rectal cancer organoids in responses to chemoradiation

Graphical abstract

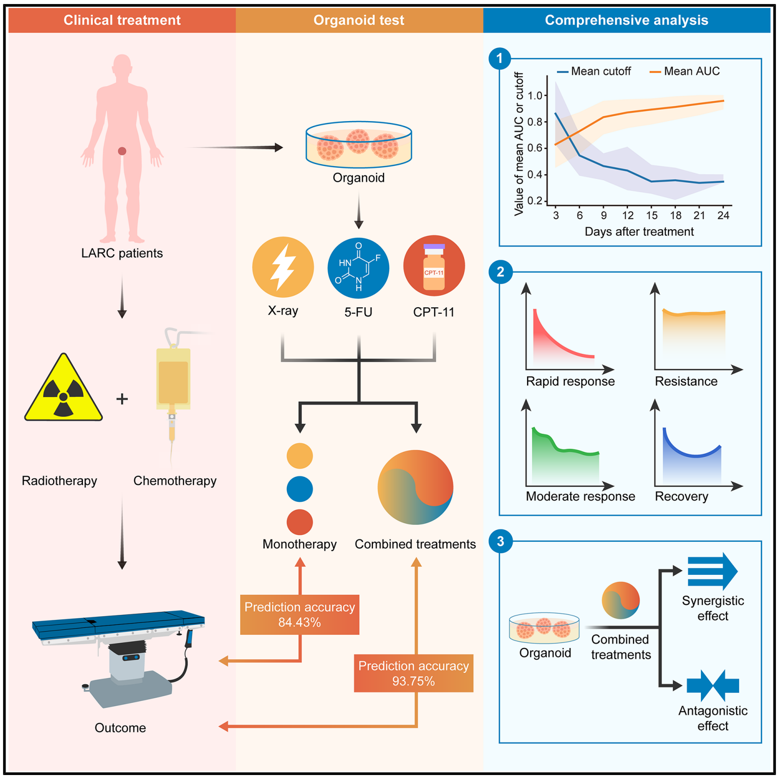

Authors

Xiaoya Xu, Tao Lv, Ye Yao, ..., Jianjun Gao, Guoqiang Hua, Zhen Zhang

Correspondence

yaoye@sysucc.org.cn (Y.Y.),
zhenzhang6@gmail.com (Z.Z.)

In brief

Xu et al. establish a rectal cancer organoid biobank and demonstrate its high accuracy in predicting clinical response, highlight time-dependent predictive accuracy of organoid assays, uncover synergistic or antagonistic effects of combined chemoradiation, and identify irinotecan as a promising neoadjuvant agent, providing valuable insights for organoid-guided precision oncology.

Highlights

- RCO responses to combined chemoradiation predict patient outcomes with 93.75% accuracy
- Organoid predictive accuracy for clinical response depends on assessment timing
- Organoid responses to combined treatment show both synergistic and antagonistic effects
- Organoids identify irinotecan as a candidate neoadjuvant agent for LARC patients

Xu et al., 2025, Cell Reports Medicine 6, 102397
October 21, 2025 © 2025 The Authors. Published by Elsevier Inc.
https://doi.org/10.1016/j.xcrm.2025.102397

CellPress

Cell Reports Medicine

CellPress
OPEN ACCESS

Article

Comprehensive dissection of rectal cancer organoids in responses to chemoradiation

Xiaoya Xu,[1,11] Tao Lv,[3,4,11] Ye Yao,[2,3,4,11,*] Juefeng Wan,[3,4] Lijun Shen,[3,4] Fan Xia,[3,4] Xiaoxue Gao,[1] Yuanchuang Li,[1] Guoxiang Fu,[1] Yun Deng,[3,4,5] Mengxue Pan,[1] Qiang Guo,[1] Xinxin Rao,[1,3,4] Peiyuan Tang,[1] Xiaomeng Li,[1] Yi Zhou,[1] Liping Liang,[3,4,6] Yaqi Wang,[3,4] Jing Zhang,[3,4] Hui Zhang,[3,4] Guichao Li,[3,4] Min Chen,[7] Junjie Peng,[8,9] Sanjun Cai,[8,9,10] Jianjun Gao,[1] Guoqiang Hua,[3,4,5] and Zhen Zhang[3,4,12,*]

[1]Institute of Radiation Medicine, Shanghai Medical College, Fudan University, Shanghai 200032, China
[2]Department of Radiation Oncology, State Key Laboratory of Oncology in South China, Guangdong Provincial Clinical Research Center for Cancer, Sun Yat-sen University Cancer Center, Guangzhou 510060, China
[3]Department of Radiation Oncology, Fudan University Shanghai Cancer Center, Fudan University, Shanghai 200032, China
[4]Department of Oncology, Shanghai Medical College, Fudan University, Shanghai 200032, China
[5]Cancer Institute, Fudan University Shanghai Cancer Center, Shanghai 230032, China
[6]Radiation Oncology Center, Huashan Hospital, Fudan University, Shanghai 200040, China
[7]APExBIO Technology LLC, Shanghai 200438, China
[8]Department of Colorectal Cancer, Fudan University Shanghai Cancer Center, Fudan University, Shanghai 200032, China
[9]Department of Oncology, Shanghai Medical College, Fudan University, Shanghai 200032, China
[10]Cancer Institute, Fudan University Shanghai Cancer Center Fudan University, Shanghai 200032, China
[11]These authors contributed equally
[12]Lead contact
*Correspondence: yaoye@sysucc.org.cn (Y.Y.), zhenzhang6@gmail.com (Z.Z.)
https://doi.org/10.1016/j.xcrm.2025.102397

SUMMARY

The responses of cancer organoids to treatments *in vitro* can predict the clinical outcomes of corresponding patients. Here, we further uncover that the responses of cancer organoids to combined treatments predict the clinical outcomes of locally advanced rectal cancer (LARC) patients more faithfully than monotherapy with 92.50% and 93.75% accuracy in the discovery and validation cohorts, respectively, which is partly due to the synergistic (3.91%, 5/128) and antagonistic (4.69%, 6/128) treatment interactions in combined chemoradiation. In addition, our detailed analysis reveals that the time point for collecting survival data of organoids is critical to the efficacy of organoids' responses to treatments for predicting the clinical outcomes of LARC patients. Besides, our current data favor the addition of irinotecan for neoadjuvant treatment of LARC patients. The comprehensive dissection of rectal cancer organoids in responses to chemoradiation here may have significant implications for the broad application of organoids in cancer precision treatments.

INTRODUCTION

Organoids are self-organized three-dimensional cultures that resemble the cellular composition, architecture, and functionality of the tissues from which they originate.[1,2] Patient-derived cancer organoids (PDCOs) closely recapitulate the histopathological and genetic features of corresponding tumors.[3,4] The responses of cancer organoids to treatments *in vitro* can faithfully predict the clinical outcomes of patients.[4–6] Nowadays, more prospective interventional randomized controlled clinical trials are ongoing by using cancer organoids to guide the treatments of patients with pancreatic cancer (ClinicalTrials.gov identifier NCT04931381 and NCT04931394) and rectal cancer (NCT05352165).

We generated a living rectal cancer organoid (RCO) biobank with 80 samples from patients with locally advanced rectal cancer (LARC) and demonstrated that PDCOs could predict chemoradiation responses of LARC.[4] In our previous study, we used response data of organoids to single treatments other than combined chemoradiation to match with clinical outcomes of LARC patients. However, LARC patients received comprehensive neoadjuvant concurrent chemoradiotherapy. In addition, the results were generated with Youden's index and bootstrapping method in a single cohort and not further validated in a larger validation cohort. And, as with several other proof-of-concept studies,[5,7,8] the response data of cancer organoids were not dissected comprehensively. Here, we reported the response data of RCOs to both single and combined treatments in an expanded cohort (128 cases), validated the efficacy of organoids to predict clinical responses of LARC patients in an independent cohort (48 cases), and provided further in-depth analysis of organoids and clinical data.

RESULTS

Optimization of rectal cancer organoid biobanking

Colorectal cancer organoids (CRCOs) were first to be established, and the culture system of CRCOs was the most developed of all tumor types.[9,10] Yet the culture efficacy of CRCOs

© 2025 The Authors. Published by Elsevier Inc.
This is an open access article under the CC BY license (http://creativecommons.org/licenses/by/4.0/).

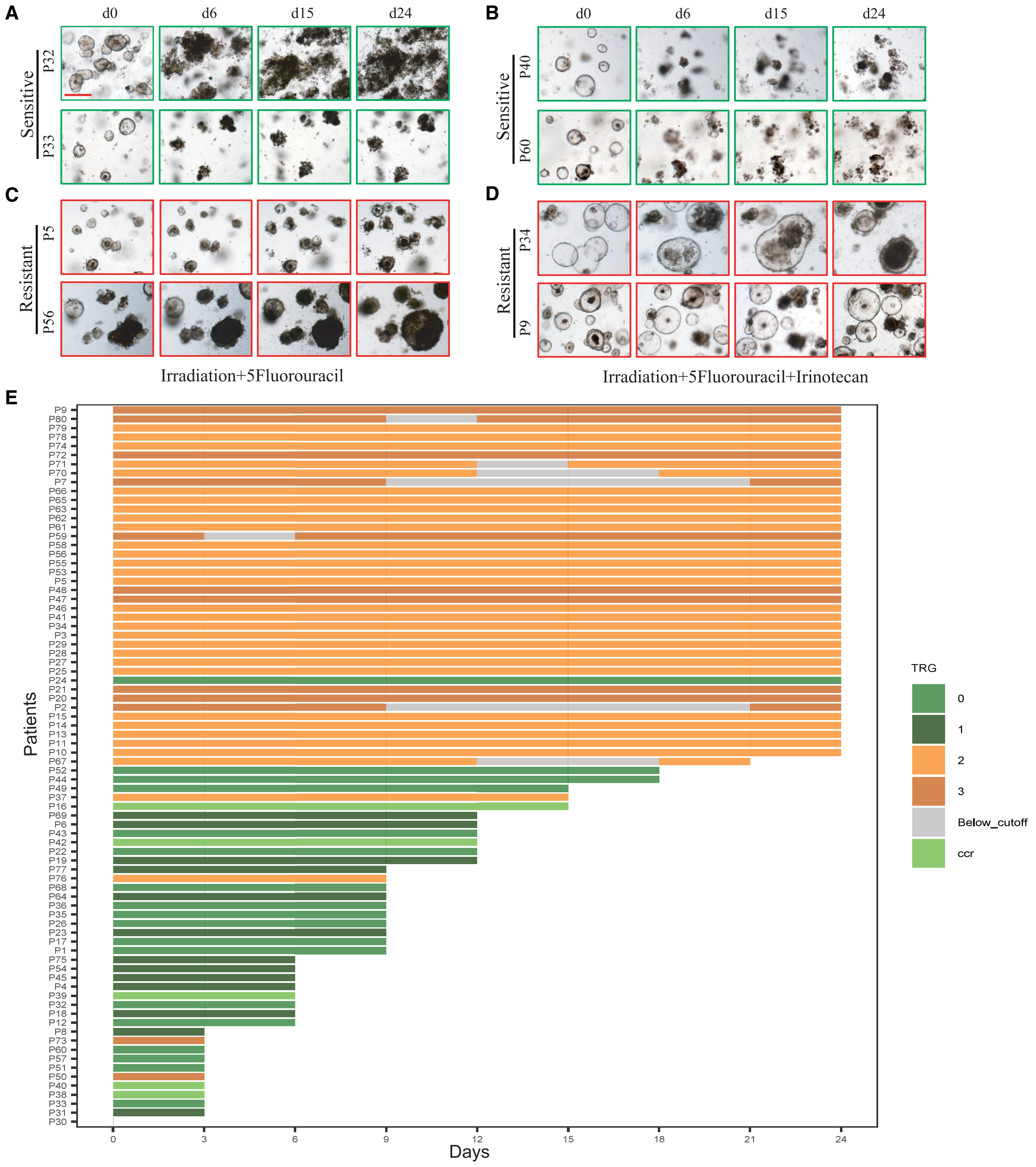

Figure 1. The responses of rectal cancer organoids to combined chemoradiation in discovery cohort with 80 cases

(A–D) Representative bright-field images of organoids after combined chemoradiation in 8 selected cases. Organoids of cases 32, 33, 40, and 60 were sensitive to combined chemoradiation. Organoids of cases 5, 9, 34, and 56 were resistant to combined chemoradiation. Organoids of cases 5, 32, 33, and 56 were treated with irradiation (8 Gy, X-rays) and 5-Fu (10 μM). Organoids of cases 9, 34, 40, and 60 were treated with irradiation (8 Gy, X-rays), 5-Fu (10 μM), and CPT-11 (10 μM). Viable organoids exhibited intact and complete structures, whereas non-viable organoids displayed disrupted and fragmented morphology. Scale bars, 200 μm.
(E) Swimmer plot illustrating temporal responses of organoids to combined chemoradiation, clustered by clinical outcomes of LARC patients. Each horizontal bar represents one patient-derived rectal cancer organoid line (n = 80). The x axis indicates the time points after combined treatment (day 0, 3, 6, 9, 12, 15, 18, 21, and 24). The y axis represents individual cases, which are grouped and ordered based on their corresponding clinical response categories: complete clinical response

(legend continued on next page)

varied, approximately ranging from 60% to 90% among different groups.[4,7,8,11,12] CRCOs recapitulate the individual diversity of colorectal cancer (CRC),[13,14] and niche factor requirements of CRCOs are heterogeneous.[15] We successfully established 96 organoid lines out of 112 rectal cancer biopsy samples (85.7%) in our previous experiences.[4] The size of tumor tissue obtained by endoscopic forceps, bacterial contamination, and culture medium were key limiting factors for the establishment of RCOs before. After that we continued optimizing the organoid culture system of rectal cancer. To enhance organoid establishment, we optimized key procedural steps, including standardized tissue collection by experienced endoscopists, antibiotic supplementation of preservation and washing solutions, controlled tissue digestion (15–20 min with intermittent shaking), minimized mechanical disruption, and completion of all processing within 90 min. In addition, we applied two parallel culture media systems (commercial and homemade) and delayed the first passaging to 9–12 days post-seeding to maximize organoid viability and expansion. And now, 142 RCO lines (87.12%, 142 of 163) were generated and expanded stably in all. Among the latter 51 biopsy samples (113–163), 46 RCO lines (90.20%, 46 of 51) were established. The success rate of RCO culture increased by 4.50% compared with before. Yet five RCOs did not expand in the culture medium used. Statistical analysis using a chi-squared test indicated that this improvement was not statistically significant (p = 0.4284). Nevertheless, the observed trend suggested that the protocol optimizations contributed positively to the overall establishment efficiency.

We reported 80 organoid lines (O1–O80) with both data of drug/irradiation sensitivity and clinical responses of corresponding patients and 11 organoid lines (O81–O91) with corresponding patients waiting for surgery and following pathological tumor regression grade (pTRG) evaluation in our earlier research.[4] Eight (O81–O85, O88, and O90–O91) of those 11 patients had received surgery, and the other 3 patients (O86–O87 and O89) reached sustained clinical complete response (cCR) and refused to undergo surgery. Among the latter generated 46 organoid lines, 32 (O92–O94, O96–O101, O103–O104, O106, and O109–O128) corresponding patients underwent surgery, and their pTRG scores were available. Five (O95, O102, O105, and O107–O108) patients had sustained cCR without surgery following concurrent chemoradiation. One patient developed distant metastases. One patient was lost to follow-up. There are still 7 patients waiting for surgery and pTRG evaluation. Hence, at the time of manuscript writing, 128 organoid lines were used for further investigation.

RCO responses to single treatments and combined chemoradiation

In our previous study, we tested the responses of RCO (O1–O91) to 5-fluorouracil (5-Fu), irinotecan (CPT-11), and irradiation separately.[4] In clinic, LARC patients from our phase 3 clinical trial (CinClare, NCT 02605265) received a combined treatment of capecitabine with/without CPT-11 neoadjuvant chemoradiotherapy. Accordingly, we subsequently tested the response of RCO lines to individual treatments (O92–O128) and combined chemoradiation (O1–O128). The clinical data of these 128 patients were presented in Table S1. Dynamic organoid size change, measuring the size of organoids after treatments, served as a measure of organoid survival ratio. This assay has been validated by direct comparison with the benchmark of organoid sensitivities test CellTiter-Glo 3D cell viability method (Promega, G9683).[4] The detailed data of RCO line (O1–O128) size change after single treatments and combined chemoradiation were summarized in Figures S1–S4 and Table S2.

In our previous research, 36.42% (95% confidence interval [CI], 26.87%–45.52%) was defined as the mean optimal cutoff value of the RCO size change ratios on day 24 to day 0 after treatments by using the data of pTRG (patient 1–patient 80, P1–P80) and RCO (O1–O80) responses to single treatments based on Youden's index and bootstrap sampling. In this study, previous 80 patients (P1–P80) and latter 48 patients (P81–P128) were served as the discovery and validation cohorts, respectively. The update optimal cutoff value of the RCO size change ratios (O1–O80) on day 24 to day 0 after combined chemoradiation was 34.87% (95% CI, 34.30%–40.14%). In the discovery cohort (O1–O80), 41 organoid lines (size recovery ratios $\leq$ 34.87%) were sensitive and the other 39 lines (size recovery ratios > 34.87%) were resistant to combined chemoradiation (Figures 1, 2, and S1–S4; Table S2). In the validation cohort (O81–O128), 20 organoid lines (size recovery ratios $\leq$ 34.87%) were sensitive and the other 28 lines (size recovery ratios > 34.87%) were resistant to combined chemoradiation (Figures 2 and S4; Table S2).

RCO responses to combined chemoradiation predict clinical outcomes of LARC patients with 93.75% accuracy

The pTRG evaluation after total mesorectal excision (TME) is the gold standard to assess the responses of rectal cancer tumors to neoadjuvant concurrent chemoradiation (NACR).[16] We defined the patients of LARC with pTRG = 0 or 1 as sensitive to NACR and others with pTRG = 2 or 3 as resistant to NACR according to the survival benefit of LARC patients with different pTRG.[16] Besides, a minority of LARC patients who achieved a sustained complete clinical response adopted a ''watch and wait'' strategy and did not receive radical surgery in our phase 3 clinical trials. These patients with sustained cCR were also considered to be sensitive to NACR. Thus, 58 LARC patients were sensitive to NACR and 70 patients were resistant to NACR (Table S1).

Chemoradiation responses in LARC patients (P1–P80) were highly matched to RCO responses to single treatment, with 84.43% accuracy, 78.01% sensitivity, and 91.97% specificity.[4] The area under the curve (AUC) was 0.882 (95% CI, 0.765–0.987).[4] LARC patients from our phase 3 clinical trial (CinClare,

(cCR), TRG = 0, TRG = 1, TRG = 2, and TRG = 3. Bars extend from day 0 until the time point before which the ratio of organoid area (relative to day 0) falls below the predefined sensitivity cutoff (0.3487), indicating a treatment-sensitive response. In rare cases, organoid regrowth was observed after initial regression, and the bar reappears at later time points when the area ratio exceeded the cutoff again. This plot captures the heterogeneity and dynamic patterns of treatment responses among organoid lines and their alignment with clinical outcomes. P indicates the patient, and O indicates the corresponding organoid line derived from that patient, with matching numbers denoting one-to-one correspondence. See also Figures S1–S4 and S7 and Tables S1 and S2.

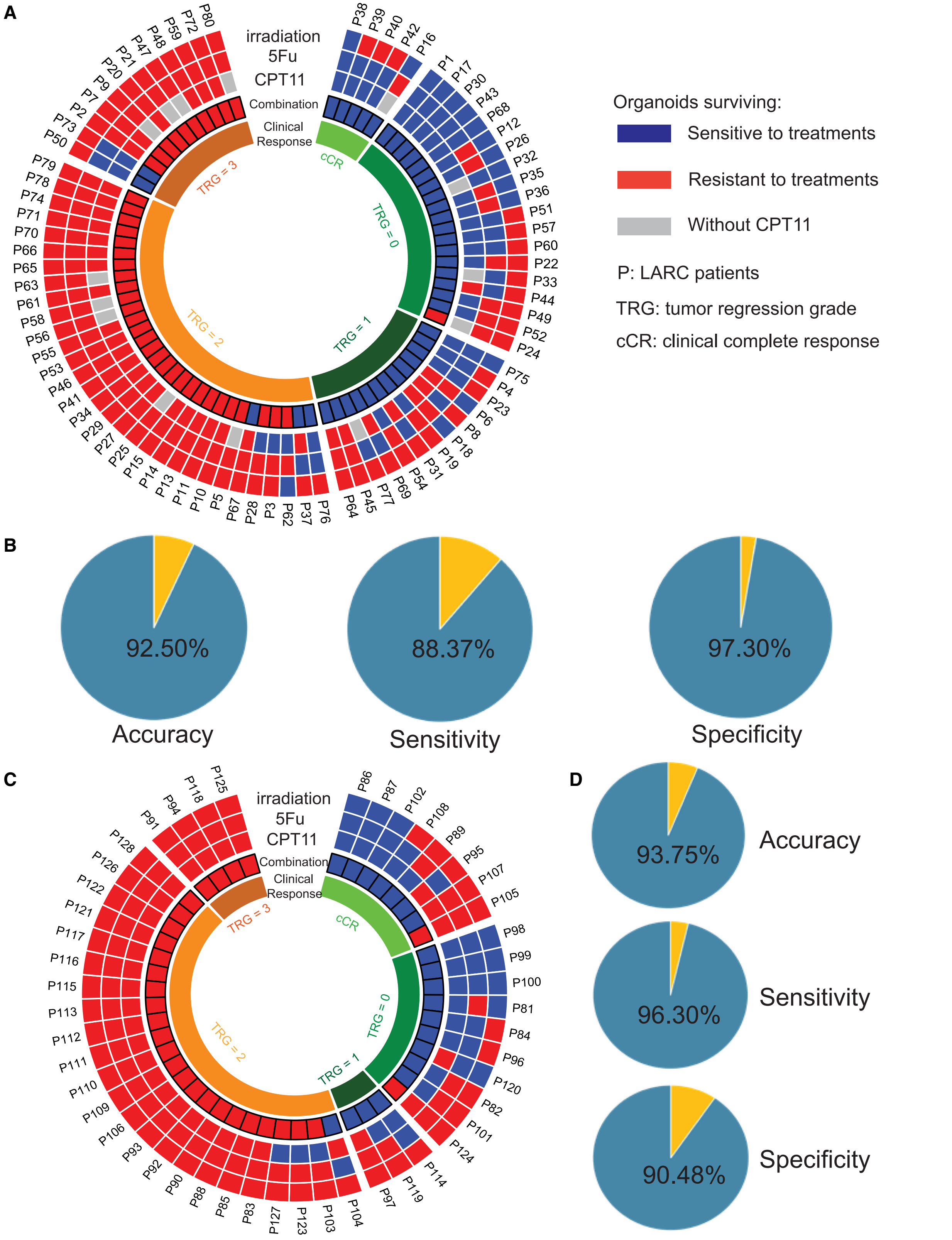

(legend on next page)

NCT 02605265) were treated with combined chemoradiation. Studies suggested that there was strong evidence of synergism between 5-Fu and radiation,[17–19] oxaliplatin and radiation,[19] and 5-fu/oxaliplatin[20] in CRC. Hence, we hypothesized that RCO responses to combined chemoradiation would have better predictive power for clinical outcomes of LARC patients than single treatments. By analyzing the data of RCO responses to combined chemoradiation and updated cutoff value (34.87%), we found that 36 patients with their organoids being sensitive to combined chemoradiation achieved good responses after NACR treatment (pTRG 0 or 1 or cCR), except for five patients (P37, P50, P67, P73, and P76) with poor responses (pTRG 2 or 3) in the discovery cohort. Among 39 patients whose organoids were resistant to combined chemoradiation, 38 cases had poor responses to NACR (TRG 2 or 3) and one patient (P24) had a good response (Figures 1E, 2A, and S1–S4; Tables S1 and S2). Next, we matched the organoid sensitivity data of combined chemoradiation with patient clinical outcomes. The results showed that chemoradiation responses in patients were highly matched to RCO responses to combined chemoradiation, with 92.5% accuracy, 88.37% sensitivity, and 97.30% specificity (Figure 2B; Table S3). The AUC was 0.958 (95% CI, 0.890–1.000).

The responses of RCO to single and combined treatments were consistent in most of the patients (92.50%, 74/80) refereed to the cutoff value (34.87%) in the discovery cohort (Figures 2A and S1–S4; Tables S1 and S2). Three organoid lines (O45, O64, and O67) were resistant to single treatments but sensitive to combined chemoradiation. Two corresponding patients (P45 and P64) had good clinical outcomes (pTRG 1), and the other patient (P67) got poor clinical response (pTRG 2). Besides, three organoid lines (O3, O28, and O62) were sensitive to single treatments (irradiation, 5-Fu, or irinotecan) but resistant to combined chemoradiation. All of the corresponding three patients got poor clinical outcomes (TRG 2 or 3) (Figures 2A and S1–S3; Tables S1 and S2). Our latest data suggested that the efficacy of RCO responses to combined chemoradiation (irradiation + 5-Fu + irinotecan) to predict clinical outcomes of LARC patients significantly improved compared with that to single treatments (irradiation/5-Fu/irinotecan) by using Pearson's chi-squared test in the discovery cohort ($p < 0.00001$) (Table S4).

Next, we further tested the predictive power of RCO responses to combined chemoradiation for clinical outcomes of LARC patients in our validation cohort with 48 samples (P81–P128). Among the 20 patients whose organoids were sensitive to combined chemoradiation, 19 cases had a good response to NACR (pTRG 0 or 1 or cCR) and only one (P104) had a poor response (pTRG 2). And 26 patients with their organoids being resistant to combined chemoradiation achieved poor responses after NACR treatment (TRG 2 or 3), except for two patients (P105 and P124) with good responses (cCR or pTRG 0) (Figures 2C and S4; Tables S1 and S2). Diagnostic test revealed that chemoradiation responses in patients of validation cohorts (P81–P128) also highly matched to RCO responses to combined chemoradiation, with 93.75% accuracy, 96.30% sensitivity, and 90.48% specificity (Figure 2D; Table S5). We also tested the efficacy of RCO responses to single treatments to predict clinical outcomes of LARC patients in the validation cohort. The results showed that the accuracy, sensitivity, and specificity rates were 83.33%, 85.19%, and 80.95%, respectively (Table S6). Further analysis also suggested that the efficacy of RCO responses to combined chemoradiation to predict clinical outcomes of LARC patients significantly improved compared with that to single treatments in the validation cohort ($p < 0.00001$) (Table S7).

Evaluation of the optimal time point for collecting response data of organoids and the diversity of organoid responses

Clinically a short time span ranging from the collection of patient tumor tissues to the final output of organoid responses to treatments matters. Therefore, delayed time point for collecting survival data of organoids after initial treatments (day 0) was detrimental to LARC patients. We used the response data of organoids at day 24 after treatments to match with clinical outcomes of LARC patients before[4] and in the current study. Here, we further investigated the efficacy of organoid response data at day 3, 6, 9, 12, 15, 18, and 21 after treatments to predict clinical outcomes of LARC patients. By using Youden's index and bootstrap sampling methods, the mean optimal cutoff values of the ratios of RCO size change (O1–O80) on day 3, 6, 9, 12, 15, 18, 21, and 24 to that on day 0 after combined chemoradiation were 85.72% (95% CI, 63.24%–110.21%), 54.97% (95% CI, 39.58%–70.48%), 46.61% (95% CI, 36.15%–56.02%), 43.36% (95% CI, 28.63%–60.97%), 34.94% (95% CI, 25.96%–47.32%), 36.00% (95% CI, 21.31%–45.42%), 33.78% (95% CI, 27.68%–40.30%), and 34.87% (95% CI, 34.30%–40.14%), respectively. This result showed that the mean optimal cutoff values gradually decreased as the time point for collecting survival data of organoids was delayed (Figure 3A). In addition, the optimal cutoff values on day 15, 18, 21, and 24 were extremely similar (Figure 3A) and indicated that the determination of organoid responses to treatments would be very close by using these four different but comparable cutoffs.

The AUCs of different time points (day 3, 6, 9, 12, 15, 18, 21, and 24 after combined chemoradiation) were 0.6268 (95% CI, 0.4600–0.7908), 0.7326 (95% CI, 0.5692–0.8810), 0.8338 (95% CI,

Figure 2. Responses of rectal cancer organoids to combined chemoradiation to predict clinical outcomes of LARC patients significantly improved than single treatments in both discovery and validation cohorts

(A) Heatmap of patients' clinical outcomes and corresponding responses of organoids to irradiation, 5-Fu, CPT-11, and combined chemoradiation in the discovery cohort with 80 cases.

(B) Results of diagnostic test (accuracy, sensitivity, and specificity) of the organoid responses to combined chemoradiation to predict patients' clinical outcomes in discovery cohort.

(C) Heatmap of patients' clinical outcomes and corresponding responses of organoids to irradiation, 5-Fu, CPT-11, and combined chemoradiation in the validation cohort with 48 cases.

(D) Results of diagnostic test (accuracy, sensitivity, and specificity) of the organoid responses to combined chemoradiation to predict patients' clinical outcomes in the validation cohort. See also Figures S1–S4 and Tables S1–S7 and S9–S11.

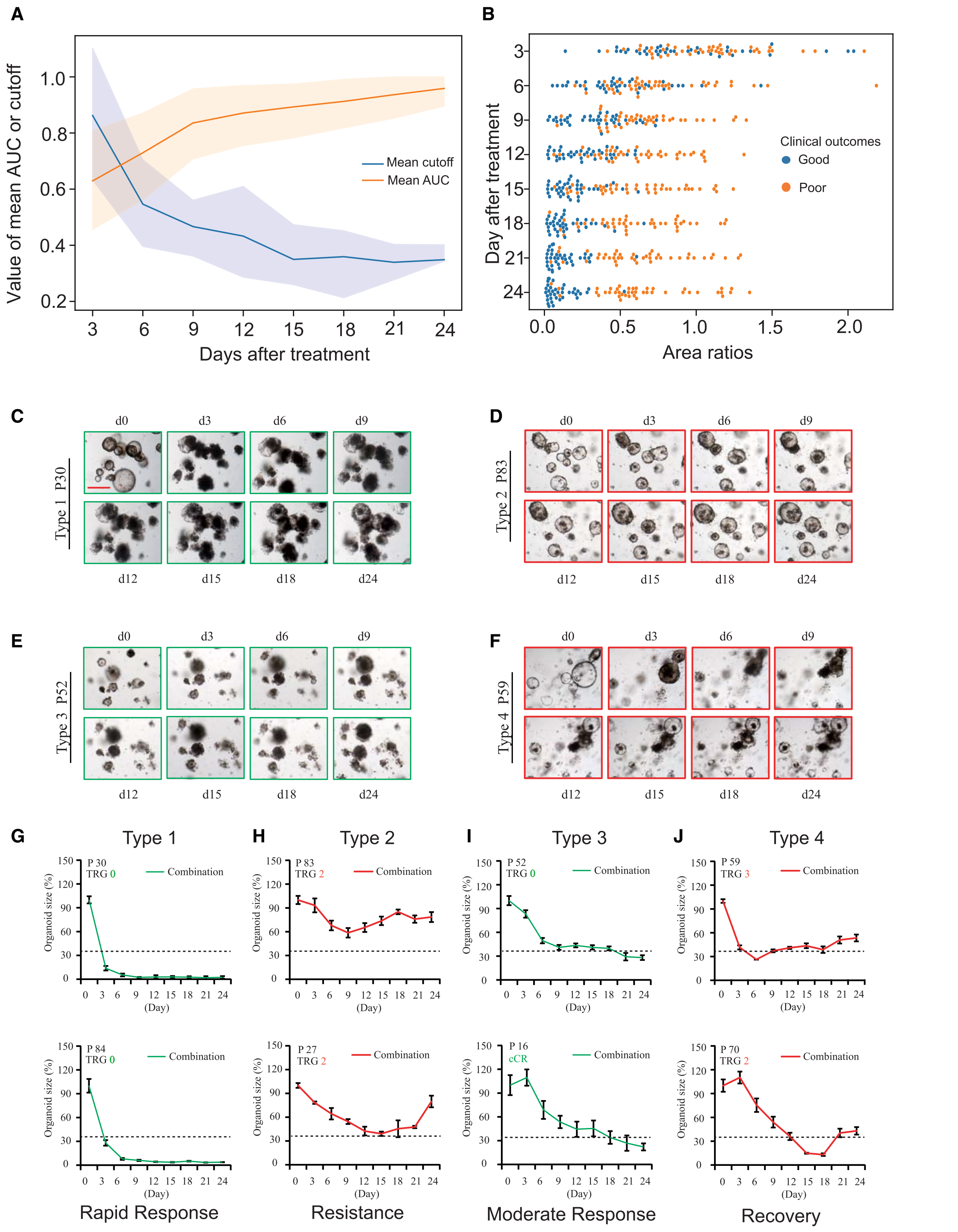

(legend on next page)

CellPress
OPEN ACCESS

0.7101–0.9454), 0.8672 (95% CI, 0.7552–0.9667), 0.8968 (95% CI, 0.7917–0.9765), 0.9122 (95% CI, 0.8143–0.9902), 0.9378 (95% CI, 0.8571–1.0000), and 0.9586 (95% CI, 0.8906–1.0000), respectively. It can be seen quite clearly that the AUCs gradually increased as the time point for collecting survival data of organoids was delayed, suggesting that the efficacy of RCO responses to treatments for predicting the clinical outcomes of LARC patients improved constantly as well (Figure 3A). The area ratios-time point diagram also uncovered a very basic similarity in predictive efficacy of organoids for clinical outcomes of LARC patients (Figure 3B). Dose response data of cancer 2D cells were generally collected within several days after treatments. Similarly, day 6 after treatments was widely used as the time point for collecting survival data of cancer organoids.[5,7,8,11] Our current results suggest that the survival data of cancer organoids at day 6 after treatments could not serve as a robust biomarker for predicting clinical outcomes of LARC patients (AUC = 0.7326). For the purposes of cancer translational research and organoids guided precision cancer treatment, we recommend that the time point for collecting survival data of cancer organoids should be no earlier than day 9 after treatments (AUC = 0.8337). If time allows in clinic, the time points of day 18, 21, and 24 after treatments are superior alternatives (AUC > 0.9).

Our results have demonstrated a great diversity of responses to single treatments[4] and combined chemoradiation among different RCO lines (Figures 1E, 2A, 2C, and S1–S4). Here, we further categorized RCOs into four groups based on the modes of responses to combined chemoradiation (Figures 3C–3J and S2–S4). Organoids with "rapid response" were extremely sensitive to chemoradiation, and the corresponding ratios of organoid size change quickly fell below the cutoff (34.87%) within 12 days after the treatment (Figures 3C, 3G, and S2). By contrast, "resistance" organoids were highly resistant to chemoradiation, and the ratios always remained above the cutoff (Figures 3D, 3H, and S4). Besides, organoids with "moderate response" were moderately sensitive to chemoradiation, and the ratios fell below the cutoff after 12 days (Figures 3E, 3I, and S3). Interestingly enough, the ratios of some organoid lines fell below the cutoff at earlier time point but then raised above the cutoff (Figures 3F, 3G, and S3). These organoid lines had capacity for "recovery" from the combined chemoradiation. We supposed that this phenomenon resulted from the intratumoral heterogeneity, which was a hallmark of cancer and had great impact on sensitivity to treatment.[21] At the early stage after treatments, the reduction of the ratios was caused by the death of sensitive organoids in these organoid lines. Then, the survived resistant organoids finally expanded and contributed to the rising of the ratios. Therefore, for the organoid lines with "moderate response" or "recovery" capacity to treatments, sufficient duration for collecting survival data to test the responses of organoids was essential. We further analyzed the relationship between the organoid response modes and the clinical outcomes of patients with LARC who received neoadjuvant chemoradiotherapy. We found that 93.6% of patients whose organoids exhibited a "rapid response" or "moderate response" pattern achieved favorable clinical outcomes, defined as TRG 0 or 1 and cCR. In contrast, 98.0% of patients whose organoids exhibited "resistance" or "recovery" patterns had poor responses to neoadjuvant therapy, corresponding to TRG 2 or 3 (Table S8). These results suggest a strong concordance between the *in vitro* organoid response patterns to combined treatments and the actual therapeutic outcomes observed in patients.

Synergistic and antagonistic treatment interactions in combined chemoradiation

Combination therapy was a cornerstone of cancer therapy. The amalgamation of cancer therapies enhances efficacy in a characteristically additive or a synergistic manner. LARC patients benefit from concurrent chemoradiation before radical surgery.[22,23] In clinic, the treatment interactions cannot be evaluated for individual LARC patients. Now this can be achieved by using the organoid system to test the responses to single treatments and combined chemoradiation. Our organoids data suggested that four LARC patients (P45, P64, P97, and P107) got good clinical outcomes (pTRG 1 and cCR) benefiting from synergistic effects among irradiation, 5-Fu, and irinotecan (Figures 2A, 2C, 4A–4D, and S2–S4; Tables S1 and S2). These four organoid lines were resistant to single treatments of irradiation, 5-Fu, and irinotecan (size recovery ratios > 34.87%) and turned to be sensitive to combined treatments (size recovery ratios < 34.87%) (Figures 2A, 2C, 4A–4D, and S2–S4; Tables S1 and S2). Exceptionally, one patient (P67) with corresponding organoid line (O67) resistant to single treatments and sensitive to combined treatments did not achieve good response after concurrent

Figure 3. Evaluation of the optimal time point for collecting survival data of rectal cancer organoids

(A) The values of mean cutoff and mean AUC by using the data of organoid response to combined chemoradiation at different time points after treatment to predict the clinical outcomes of LARC patients who underwent neoadjuvant chemoradiation in the discovery cohort (O1–O80). The ratios of organoid size change on different time points to that on day 0 after treatments served as a measure of organoid response to combined chemoradiation. The *x* axis indicates the time points for survival data collection of organoids following combined treatment.

(B) The distribution patterns of the ratios of organoid size change on different time points to those on day 0 after treatments in the discovery cohort (O1–O80). The blue and orange dots indicated that corresponding patients got good (TRG 0/1, or cCR) and poor (TRG 2/3) clinical outcomes, respectively.

(C–F) Representative bright-field images of organoids at day 0, 3, 6, 9, 12, 15, 18, and 24 after combined chemoradiation in four groups of organoids (type 1, 2, 3, and 4) categorized based on the modes of responses to treatment. Scale bars, 200 μm.

(G–J) Size change curves of organoids after combined chemoradiation in 8 selected cases. The organoids of P30 and P84 were sensitive to combined chemoradiation and had a "rapid response" after treatment (type 1). The organoids of P27 and P83 were highly resistant to chemoradiation, and the ratios always remained above the cutoff (type 2). The organoids of P16 and P52 were moderately sensitive to chemoradiation and had a "moderate response" after treatment (type 3). The ratios of organoids from P59 and P70 fell below the cutoff at earlier time point but then raised above the cutoff. Organoid of P59 and P70 had capacity for "recovery" from the combined chemoradiation (type 4). Organoid size data shown were means ± SEM from three independent experiments (n = 12). Red curves, patients with a poor clinical response (TRG 2 or 3); green curves, patients with a good clinical response (TRG 0 or 1 or cCR). Dotted lines indicated cutoff of organoid size change (34.87%). See also Figure S6 and Table S8.

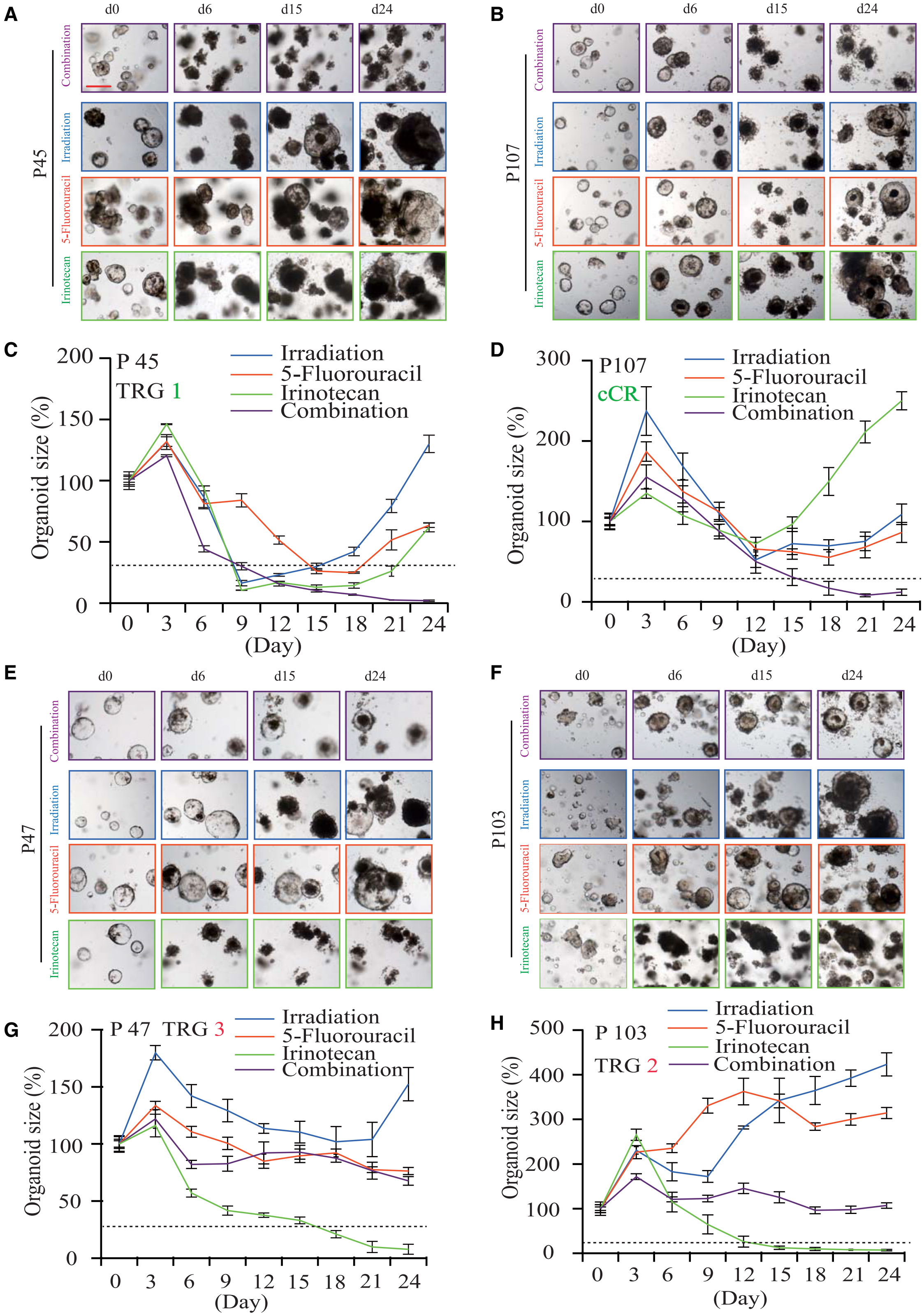

(legend on next page)

chemoradiation (pTRG 2) (Figures 2A and S3; Tables S1 and S2). In all, response data of 5 organoid lines (3.91%, 5/128) exhibited synergistic effects after combined treatments.

Personalized cancer treatments by using combinations of treatments (radiation, chemotherapy, and targeted drugs, etc.) with a synergistic effect were highly attractive. Therefore, drug synergy (at least additivity) was in the spotlight on translational and clinical research.[24,25] Yet antagonistic effect of the combined treatments on cancer also existed in *in vitro* tumor models.[26–28] In the current study, five LARC patients (P3, P28, P103, P123, and P127) (3.91%, 5/128) got poor clinical outcomes (pTRG 2 or 3) with corresponding organoid lines sensitive to irinotecan and resistant to combined chemoradiation. One LARC patient (P62) (0.78%, 1/128) had a poor clinical outcome (pTRG 2), and the organoid line of this patient was sensitive to irradiation and irinotecan but resistant to combined chemoradiation (Figures 2A, 2C, 4E–4H, and S1–S4; Tables S1 and S2). Altogether, six LARC patients (4.69%, 6/128) got poor responses probably due to antagonistic effect in combined treatments. Data from CRC cell lines had revealed that antagonistic effect was noted when CRC cells were simultaneously exposed to 5-FU and irinotecan.[28] Hence, the inhibition of irinotecan and irradiation on RCO may be hindered by 5-Fu (Figures 2A, 2C, 4E–4H, and S1–S4; Tables S1 and S2). With the exception of the 11 cases as mentioned earlier, synergistic and antagonistic effects were not observed in the other 117 organoid lines. Organoids were resistant to combined chemoradiation when they were resistant to single treatments of irradiation, 5-Fu, and irinotecan, and organoids were sensitive to combined chemoradiation when they were sensitive to at least one of the individual treatments in the other 117 cases (Figures 2A, 2C, and S5).

Organoid test suggested the potential of irinotecan for neoadjuvant treatment of LARC patients

NACR followed by TME was the standard care for LARC patients. Radiotherapy (RT) combined with 5-Fu is the main modality of NACR. Although the addition of oxaliplatin to NACR is not recommended at this time by the NCCN (National Comprehensive Cancer Network) guideline, several large randomized phase 3 trials addressed the addition of oxaliplatin to the regimens.[29] For irinotecan, it was not discussed for LARC patients in the latest version of NCCN rectal cancer guideline.[29] But the results of our phase 3 clinical trial (CinClare, NCT 02605265) showed that adding irinotecan to NACR significantly increased pathological complete response rate in LARC patients (15% vs. 30%, p = 0.001).[30] Consistent with the clinical outcome described earlier, our organoids data also suggested that irinotecan had considerable potential for the neoadjuvant treatment of LARC patients. Surprisingly, organoid test exhibited that irinotecan was fairly more effective than irradiation and 5-Fu (Figures 2A and 2C; Table S2). The rates of organoid lines sensitive to irradiation, 5-Fu, and irinotecan were 18.75% (24/128), 30.47% (39/128), and 42.11% (48/114), respectively ($p < 0.001$) (Figures 2A and 2C; Tables S2 and S9). Moreover, some organoid lines were only sensitive to just one of the treatments (irradiation/5-Fu/irinotecan) and resistant to the other two. Most of their corresponding LARC patients achieved good clinical responses and probably benefited from the treatments, which their organoid lines were sensitive to. More specifically, 1.56% (2/128) (P6 and P18), 6.25% (8/128) (P8, P31, P33, P37, P44, P69, P95, and P104), and 12.28% (14/114) (P3, P19, P22, P28, P49, P52, P54, P82, P89, P101, P103, P119, P123, and P127) of organoid lines were only sensitive to irradiation, 5-Fu, and irinotecan, respectively ($p < 0.01$) (Figures 2A and 2C; Tables S2 and S10). Among the 14 LARC patients whose organoid lines were only sensitive to irinotecan, 9 (64.29%, 9/14) of them (P19, P22, P49, P52, P54, P82, P89, P101, and P119) had good clinical responses and probably benefited from irinotecan treatment (Figures 2A and 2C; Tables S1 and S2). Earlier analysis favored the addition of irinotecan in neoadjuvant treatment of LARC patients. Further analysis of long-term follow-up results of CinClare clinical trial combined with the sensitivity data of corresponding cancer organoids showed that the LARC patients with corresponding organoids sensitive to irinotecan had higher rates of 3-year disease-free survival (71.6% vs. 55.5%, p = 0.034) and distant metastasis-free survival (77.9% vs. 57.2%, p = 0.015) than the patients with corresponding organoids resistant to irinotecan. On the contrary, the sensitivity of LARC cancer organoids to 5-FU and irradiation failed to predict 3-year disease-free survival (5-FU: 65.4% vs. 61.9%, p = 0.643; irradiation: 84.8% vs. 57.8%; p = 0.072).[31] Our organoid data await further validation of more phase 3 clinical trials, including the 5-year follow-up results of CinClare.

DISCUSSION

Our findings uncovered that responses of cancer organoids to combined treatments predicted the clinical outcomes of corresponding LARC patients more faithfully with 93.75% accuracy than single treatments, which was partly due to the synergistic and antagonistic treatment interactions in combined chemoradiation. Hence, when organoids are used to guide the precision treatment of cancer patients, both single and feasible combined treatments *in vitro* should be tested to nominate optimal combination of therapeutic agents for patients. Combination of therapies, which were individually ineffective, possibly will lead to

Figure 4. Synergistic and antagonistic treatment interactions in combined chemoradiation

(A and B) Representative bright-field images of two selected organoid lines (O45 and O107), which were resistant to single treatments of irradiation, 5-Fu, and CPT-11 but sensitive to combined chemoradiation at day 0, 6, 15, and 24 after treatments. Scale bars, 200 μm.

(C and D) Size change curves of organoids after single treatments and combined chemoradiation in 2 selected cases (O45 and O107).

(E and F) Representative bright-field images of two selected organoid lines (O47 and O103), which were sensitive to irinotecan but resistant to combined chemoradiation at day 0, 6, 15, and 24 after treatments.

(G and H) Size change curves of organoids after single treatments and combined chemoradiation in 2 selected cases (O47 and O103). Organoid size data shown were means ± SEM from three independent experiments (n = 12). Red curves, patients with a poor clinical response (TRG 2 or 3); green curves, patients with a good clinical response (TRG 0 or 1 or cCR). Dotted lines indicated cutoff of organoid size change (34.87%). See also Figures S5 and S7 and Table S11.

better clinical outcomes owing to synergistic effects (P45, P64, P97, and P107). On the contrary, patients could become resistant to the combinations with individually effective treatments because of antagonistic effects (P3, P28, P47, P58, P62, P103, P123, and P127). When simply integrating several individually effective agents as the therapeutic regimen without testing, the optimal combined treatments are likely to be missed out and the tumors of cancer patients may be resistant to the treatments because of the synergistic and antagonistic effect, respectively.

Clinical outcomes of cancer patients are always the output of comprehensive treatment, including RT, chemotherapy, targeted therapy, immunotherapy, etc.[32,33] Cancer organoids can be used *in vitro* for evaluating the efficacy of individual and combined treatments. Our current data revealed synergistic and antagonistic effects in combined chemoradiation and favored the addition of irinotecan for neoadjuvant treatment of LARC patients. These insights exhibited the ability of cancer organoids to optimize treatment regimen for LARC patients. These results also suggested that oncologists can be inspired to design clinical trials based on the results of cancer organoid test *in vitro*. Compared with previous research,[4,5,7,8] our current study yielded comprehensive and detailed dissection of RCOs in responses to chemoradiation including but not limited to the efficacy of organoids for predicting the clinical outcomes of LARC patients.

Wnt signaling pathway was altered in approximately 93% of all CRC tumors,[34] allowing them to grow independently of exogenous Wnt ligands *in vitro*. The success rate of RCO establishment is of great significance clinically and can be theoretically close to 93% by using culture medium without containing Wnt ligand. We and Clevers H. group[11] reported a success rate of approximately 90% for culturing CRCOs. We suggested that the CRCO culture system should be carefully optimized based on the principles and practices from our and other groups[11,15] to improve the culture efficacy. Based on the biological feature, we employed a Wnt3a-free culture system to selectively promote the outgrowth of tumor organoids while limiting the expansion of normal colonic epithelium, which is otherwise highly dependent on Wnt3a and proliferates more rapidly. To further increase the success rate, particularly for the minority of tumors that retain Wnt ligand dependency, we propose a two-step culture approach: initiating organoid culture in Wnt3a-free medium to suppress normal epithelial cells, followed by switching a portion of wells to Wnt3a-containing medium after 6–9 days. This strategy may enhance the capture of tumor subtypes with intact Wnt signaling while minimizing contamination by normal epithelium. Future studies are warranted to optimize this protocol and improve the inclusivity of organoid models.

We also had valuable proposals about the optimal time point for collecting survival data of organoids. We noticed that some organoid lines survived well more than 6–9 days after treatments but disintegrated and died subsequently. Some lines were sensitive to treatments around 6–9 days but then began to grow gradually. Therefore, we supposed that the prediction efficacy of organoids survival data at day 6–9 after treatments was relatively poor. Our detailed analysis of diagnostic test supported this hypothesis. The efficacy of RCO responses to treatments for predicting the clinical outcomes of LARC patients improved gradually as the time point for collecting survival data of organoids was delayed. Hence, we recommended that both the time point for collecting organoid survival data and the prediction efficacy needed to be fully considered, especially when organoids were used for guiding the treatments of cancer patients. Our study revealed a continuous improvement in the predictive performance of RCO responses to chemoradiotherapy from day 3 to day 24 post-treatment, as indicated by a gradual increase in AUC values. Notably, no plateau in predictive efficacy was observed on day 24, suggesting that biologically relevant response dynamics likely extend beyond this time point (Figure S6). This observation aligns with clinical evidence indicating that the full effects of RT often emerge over prolonged periods, with tumor regression and treatment response assessments evolving up to 17–19 weeks after therapy. These delayed effects reflect the complex nature of RT-induced damage, which involves not only direct cytotoxicity but also subsequent immunomodulation, vascular, and stromal remodeling and cellular repair or recovery processes.[35–37] Surviving tumor subpopulations may undergo delayed regrowth due to intrinsic resistance mechanisms or reconstitution from a heterogeneous cellular pool, emphasizing the need for extended observation windows to fully capture these dynamics. Our time-course analysis also revealed diverse RCO response patterns—including rapid, moderate, resistant, and recovery phenotypes—further highlighting the heterogeneity of treatment sensitivity. In particular, organoids with intermediate or recovery-type responses may require longer observation to accurately reflect their therapeutic trajectory. While many studies in the field adopt a standard 6–9 day window for assessing organoid survival post-treatment, our findings suggest that this may underestimate clinically relevant responses. Importantly, we observed that day 9 marked the earliest time point with AUC > 0.8, whereas time points beyond day 18 yielded consistently robust predictive values (AUC > 0.9). These results not only demonstrate the translational value of extended RCO monitoring but also resonate with current clinical needs, where accurate early prediction of response is critical, yet delayed biological responses must still be accounted for. Our findings advocate an optimized balance between clinical feasibility and biological fidelity, reinforcing the importance of choosing appropriate time points in organoid-based modeling of RT response.

Although our organoid models indicated a higher response rate to irinotecan, this finding must be contextualized within the broader clinical evidence base. Within the neoadjuvant (and especially the total neoadjuvant) setting, the strongest evidence supports oxaliplatin-based regimens. In the current study, we did not test the responses of organoids to oxaliplatin. This study represented a translational component of the CinClare clinical trial and aimed at optimizing screening of patient-derived organoids as a tool to predict clinical outcomes of rectal cancer patients treated with neoadjuvant chemoradiation. In CinClare clinical trial, eligible patients were randomly allocated to the control group (pelvic radiation of 50 Gy/25 fractions with concurrent capecitabine) or the experimental group (radiation with capecitabine combined with weekly irinotecan). Accordingly, to ensure

consistency with CinClare clinical trial, we employed the corresponding treatment regimens in our *in vitro* organoid experiments (irradiation with 5-Fu or irradiation with 5-Fu + CPT-11). Here, we present the *in vitro* organoid testing results of 10 RCO lines with the addition of oxaliplatin (Figure S7; Table S11). Recognizing its potential relevance, we intend to further pursue this line of investigation in future work. Phase 3 trials such as ARISTOTLE[38] and the meta-analysis by Greenhalgh et al.[39] reported limited efficacy of irinotecan in enhancing treatment outcomes when added to neoadjuvant chemoradiotherapy protocols. In contrast, the CinClare trial demonstrated a significant improvement in pathological complete response with irinotecan addition[30]; however, it was conducted exclusively in an Asian cohort, and its applicability to other populations remains uncertain. These discrepancies highlight the intrinsic limitations of *in vitro* organoid models, which reflect tumor cell-intrinsic chemosensitivity but lack the influence of tumor microenvironmental factors, pharmacokinetics, and host-related variability that are critical *in vivo*. Therefore, while our findings suggest potential therapeutic value, clinical translation should proceed with caution and require further validation in diverse prospective clinical studies.

Recent clinical efforts have explored the feasibility of omitting RT in the neoadjuvant treatment of LARC to reduce toxicity, especially in selected low-risk patients. For instance, the phase 3 FOWARC trial demonstrated that neoadjuvant FOLFOX alone achieved lower rates of tumor downstaging and pathologic complete response compared to chemoradiotherapy but showed no significant difference in long-term survival outcomes such as DFS and OS.[40] In our study, organoid models were subjected to monotherapies and combination treatment, enabling a nuanced view of treatment response. Notably, a subset of organoids showed sensitivity to chemotherapy alone while being resistant to radiation or combination therapy. These cases raise the possibility that organoid profiling might help identify patients who could benefit from chemotherapy-only strategies, thereby sparing them the adverse effects of radiation. However, given the limitations of *in vitro* systems in replicating tumor-stroma and immune interactions, caution is warranted when extrapolating these findings to clinical decision-making. Future studies integrating organoid testing with radiological, pathological, and molecular risk stratification tools may provide a more reliable framework for treatment de-escalation in LARC.

Our current investigation demonstrated a superior efficacy of cancer organoids for predicting the clinical outcomes of LARC patients in the discovery and validation cohorts. Most notably, the comprehensive dissection of RCOs in responses to chemoradiation disclosed several findings, inclusive of the superiority of combined treatments for prediction accuracy, significance of time point determination for collecting survival data of organoids, and synergistic/antagonistic effects in combined treatments of organoids. These highlights may have significant implications for the use of organoids in cancer precision treatments. Together with the data here, the efficacy of organoids for predicting clinical outcomes of CRC has been well documented.[4,5,7,8] Next, more randomized clinical trials will be the focus of this area.

Limitations of the study

While organoid models faithfully capture many epithelial-intrinsic characteristics of rectal cancer and enable individualized therapeutic testing, they inherently lack key components of the tumor microenvironment , including immune cells, cancer-associated fibroblasts, and vasculature. These elements play crucial roles in modulating responses to chemoradiotherapy, particularly by shaping treatment sensitivity and mediating resistance mechanisms.[41,42] Consequently, our findings primarily reflect the epithelial compartment's contribution to treatment outcomes—an essential, yet incomplete, aspect of tumor biology. Future studies integrating coculture platforms or *in vivo* systems will be necessary to elucidate epithelial-stromal-immune interactions and further enhance the translational relevance of organoid-based predictive assays. Increasing evidence supports the therapeutic enhancement of adding oxaliplatin to total neoadjuvant therapy for LARC patients. However, our study primarily focused on the CinClare trial, which investigated the efficacy of adding irinotecan into capecitabine-based neoadjuvant chemoradiotherapy for LARC. Although we briefly present *in vitro* organoid sensitivity data for oxaliplatin-based regimens in a limited number of cases, the lack of robust clinical investigation into oxaliplatin-based regimens remains a major limitation of this work that must be acknowledged. Another limitation of our study was the lack of comprehensive molecular characterization of the tested organoid models. Although our primary goal was to predict treatment responses through direct *in vitro* functional assays, we acknowledge that molecular profiling could provide deeper mechanistic insights into differential therapeutic responses. Preliminary efforts utilizing bulk and single-cell transcriptomic analyses are underway and will be crucial for elucidating resistance mechanisms and refining predictive models in the future.

RESOURCE AVAILABILITY

Lead contact

Requests for resources and further information should be directed to and will be fulfilled by the lead contact, Zhen Zhang (zhenzhang6@gmail.com).

Materials availability

This study did not generate new unique reagents.

Data and code availability

- Figures S1–S4 and Tables S3–S10 are deposited in the Mendeley Database under the following DOI: https://doi.org/10.17632/z6mdxvzwky.1.
- This study did not generate original code. All custom scripts used for data analysis were written using existing software packages, as described in the STAR Methods section, and are available from the corresponding author upon request.
- Any additional information required to reanalyze the data reported in this work paper is available from the lead contact upon request.

ACKNOWLEDGMENTS

We thank all of the LARC patients and their families for participating in the current study. We also thank Shan He and Yajuan Xiang for daily support with biopsy affairs. This work was supported by the National Natural Science Foundation of China (31470826, 31670858, 81773357, and 82102833) and Shanghai Anticancer Association EYAS PROJECT (SACA-CY23B06).

AUTHOR CONTRIBUTIONS

Conception and design, X.X., Y.Y., G.H., and Z.Z.; development of methodology, X.X., Y.Y., T.L., G.H., and Z.Z.; acquisition of data and material, X.X., Y.Y., T.L., J.W., L.S., F.X., X.G., Y.L., G.F., Y.D., M.P., Q.G., X.R., P.T., X.L.,Y.Z., L. L., Y.W., J.Z., H.Z., G.L., J.P., S.C., and J.G.; analysis and interpretation of data, M.C., X.X., Y.Y., T.L., and Z.Z.; writing, review, and/or revision of the manuscript, X.X., T.L., Y.Y., and Z.Z.; study supervision, Y.Y. and Z.Z.

DECLARATION OF INTERESTS

G.H. is an inventor of several patents related to organoid technology, and he is the founder of D1Med, a company that focuses on the cultivation of organoid models.

STAR★METHODS

Detailed methods are provided in the online version of this paper and include the following:

- KEY RESOURCES TABLE
- EXPERIMENTAL MODEL AND STUDY PARTICIPANT DETAILS
  - Patient information and human specimens
  - Rectal cancer organoids culture medium
- METHOD DETAILS
  - Tumor cell isolation and organoid culture
  - Drug/irradiation screening
  - Criteria for defining synergism and antagonism
  - Clinical response evaluation
- QUANTIFICATION AND STATISTICAL ANALYSIS

SUPPLEMENTAL INFORMATION

Supplemental information can be found online at https://doi.org/10.1016/j.xcrm.2025.102397.

Received: December 16, 2024
Revised: April 30, 2025
Accepted: September 10, 2025
Published: October 3, 2025

REFERENCES

1. Sato, T., Vries, R.G., Snippert, H.J., van de Wetering, M., Barker, N., Stange, D.E., van Es, J.H., Abo, A., Kujala, P., Peters, P.J., and Clevers, H. (2009). Single Lgr5 stem cells build crypt-villus structures in vitro without a mesenchymal niche. Nature *459*, 262–265. https://doi.org/10.1038/nature07935.
2. Artegiani, B., and Clevers, H. (2018). Use and application of 3D-organoid technology. Hum. Mol. Genet. *27*, R99–R107. https://doi.org/10.1093/hmg/ddy187.
3. Drost, J., and Clevers, H. (2018). Organoids in cancer research. Nat. Rev. Cancer *18*, 407–418. https://doi.org/10.1038/s41568-018-0007-6.
4. Yao, Y., Xu, X., Yang, L., Zhu, J., Wan, J., Shen, L., Xia, F., Fu, G., Deng, Y., Pan, M., et al. (2020). Patient-Derived Organoids Predict Chemoradiation Responses of Locally Advanced Rectal Cancer. Cell Stem Cell *26*, 17–26.e6. https://doi.org/10.1016/j.stem.2019.10.010.
5. Vlachogiannis, G., Hedayat, S., Vatsiou, A., Jamin, Y., Fernández-Mateos, J., Khan, K., Lampis, A., Eason, K., Huntingford, I., Burke, R., et al. (2018). Patient-derived organoids model treatment response of metastatic gastrointestinal cancers. Science *359*, 920–926. https://doi.org/10.1126/science.aao2774.
6. Li, M., and Izpisua Belmonte, J.C. (2019). Organoids - Preclinical Models of Human Disease. N. Engl. J. Med. *380*, 569–579. https://doi.org/10.1056/NEJMra1806175.
7. Ganesh, K., Wu, C., O'Rourke, K.P., Szeglin, B.C., Zheng, Y., Sauvé, C.E.G., Adileh, M., Wasserman, I., Marco, M.R., Kim, A.S., et al. (2019). A rectal cancer organoid platform to study individual responses to chemoradiation. Nat. Med. *25*, 1607–1614. https://doi.org/10.1038/s41591-019-0584-2.
8. Ooft, S.N., Weeber, F., Dijkstra, K.K., McLean, C.M., Kaing, S., van Werkhoven, E., Schipper, L., Hoes, L., Vis, D.J., van de Haar, J., et al. (2019). Patient-derived organoids can predict response to chemotherapy in metastatic colorectal cancer patients. Sci. Transl. Med. *11*, eaay2574. https://doi.org/10.1126/scitranslmed.aay2574.
9. Sato, T., Stange, D.E., Ferrante, M., Vries, R.G., Van Es, J.H., Van Den Brink, S., Van Houdt, W.J., Pronk, A., Van Gorp, J., Siersema, P.D., et al. (2011). Long-term expansion of epithelial organoids from human colon, adenoma, adenocarcinoma, and Barrett's epithelium. Gastroenterology *141*, 1762–1772. https://doi.org/10.1053/j.gastro.2011.07.050.
10. Sato, T., Stange, D.E., Ferrante, M., Vries, R.G.J., Van Es, J.H., Van den Brink, S., Van Houdt, W.J., Pronk, A., Van Gorp, J., Siersema, P.D., and Clevers, H. (2011). Long-term expansion of epithelial organoids from human colon, adenoma, adenocarcinoma, and Barrett's epithelium. Gastroenterology *141*, 1762–1772. https://doi.org/10.1053/j.gastro.2011.07.050.
11. van de Wetering, M., Francies, H.E., Francis, J.M., Bounova, G., Iorio, F., Pronk, A., van Houdt, W., van Gorp, J., Taylor-Weiner, A., Kester, L., et al. (2015). Prospective derivation of a living organoid biobank of colorectal cancer patients. Cell *161*, 933–945. https://doi.org/10.1016/j.cell.2015.03.053.
12. Schütte, M., Risch, T., Abdavi-Azar, N., Boehnke, K., Schumacher, D., Keil, M., Yildiriman, R., Jandrasits, C., Borodina, T., Amstislavskiy, V., et al. (2017). Molecular dissection of colorectal cancer in pre-clinical models identifies biomarkers predicting sensitivity to EGFR inhibitors. Nat. Commun. *8*, 14262. https://doi.org/10.1038/ncomms14262.
13. Weeber, F., van de Wetering, M., Hoogstraat, M., Dijkstra, K.K., Krijgsman, O., Kuilman, T., Gadellaa-van Hooijdonk, C.G.M., van der Velden, D.L., Peeper, D.S., Cuppen, E.P.J.G., et al. (2015). Preserved genetic diversity in organoids cultured from biopsies of human colorectal cancer metastases. Proc. Natl. Acad. Sci. USA *112*, 13308–13311. https://doi.org/10.1073/pnas.1516689112.
14. Cristobal, A., van den Toorn, H.W.P., van de Wetering, M., Clevers, H., Heck, A.J.R., and Mohammed, S. (2017). Personalized Proteome Profiles of Healthy and Tumor Human Colon Organoids Reveal Both Individual Diversity and Basic Features of Colorectal Cancer. Cell Rep. *18*, 263–274. https://doi.org/10.1016/j.celrep.2016.12.016.
15. Fujii, M., Shimokawa, M., Date, S., Takano, A., Matano, M., Nanki, K., Ohta, Y., Toshimitsu, K., Nakazato, Y., Kawasaki, K., et al. (2016). A Colorectal Tumor Organoid Library Demonstrates Progressive Loss of Niche Factor Requirements during Tumorigenesis. Cell Stem Cell *18*, 827–838. https://doi.org/10.1016/j.stem.2016.04.003.
16. Trakarnsanga, A., Gönen, M., Shia, J., Nash, G.M., Temple, L.K., Guillem, J.G., Paty, P.B., Goodman, K.A., Wu, A., Gollub, M., et al. (2014). Comparison of tumor regression grade systems for locally advanced rectal cancer after multimodality treatment. J Natl Cancer Inst *106*, dju248. https://doi.org/10.1093/jnci/dju248.
17. Hall, B.E., and Good, J.W. (1962). Treatment of far-advanced cancer with 5-fluorouracil, used alone and in combination with irradiation. Incidence and duration of remission and survival-time data in 223 patients. Cancer Chemother. Rep. *16*, 369–386.
18. Millington, E. (1966). The combined treatment of carcinoma of the rectum with cobalt and chemotherapy. Am. J. Roentgenol. Radium Ther. Nucl. Med. *96*, 635–639. https://doi.org/10.2214/ajr.96.3.635.
19. Kjellström, J., Kjellén, E., and Johnsson, A. (2005). In vitro radiosensitization by oxaliplatin and 5-fluorouracil in a human colon cancer cell line. Acta Oncol. *44*, 687–693. https://doi.org/10.1080/02841860500247552.
20. deBraud, F., Munzone, E., Nolè, F., De Pas, T., Biffi, R., Brienza, S., and Aapro, M.S. (1998). Synergistic activity of oxaliplatin and 5-fluorouracil in

CellPress
OPEN ACCESS

patients with metastatic colorectal cancer with progressive disease while on or after 5-fluorouracil. Am. J. Clin. Oncol. *21*, 279–283. https://doi.org/10.1097/00000421-199806000-00015.

21. Vitale, I., Shema, E., Loi, S., and Galluzzi, L. (2021). Intratumoral heterogeneity in cancer progression and response to immunotherapy. Nat. Med. *27*, 212–224. https://doi.org/10.1038/s41591-021-01233-9.
22. Sauer, R., Becker, H., Hohenberger, W., Rödel, C., Wittekind, C., Fietkau, R., Martus, P., Tschmelitsch, J., Hager, E., Hess, C.F., et al. (2004). Preoperative versus postoperative chemoradiotherapy for rectal cancer. N. Engl. J. Med. *351*, 1731–1740. https://doi.org/10.1056/NEJMoa040694.
23. Rödel, C., Graeven, U., Fietkau, R., Hohenberger, W., Hothorn, T., Arnold, D., Hofheinz, R.D., Ghadimi, M., Wolff, H.A., Lang-Welzenbach, M., et al. (2015). Oxaliplatin added to fluorouracil-based preoperative chemoradiotherapy and postoperative chemotherapy of locally advanced rectal cancer (the German CAO/ARO/AIO-04 study): final results of the multicentre, open-label, randomised, phase 3 trial. Lancet Oncol. *16*, 979–989. https://doi.org/10.1016/s1470-2045(15)00159-x.
24. Narayan, R.S., Molenaar, P., Teng, J., Cornelissen, F.M.G., Roelofs, I., Menezes, R., Dik, R., Lagerweij, T., Broersma, Y., Petersen, N., et al. (2020). A cancer drug atlas enables synergistic targeting of independent drug vulnerabilities. Nat. Commun. *11*, 2935. https://doi.org/10.1038/s41467-020-16735-2.
25. Palmer, A.C., and Sorger, P.K. (2017). Combination Cancer Therapy Can Confer Benefit via Patient-to-Patient Variability without Drug Additivity or Synergy. Cell *171*, 1678–1691.e13. https://doi.org/10.1016/j.cell.2017.11.009.
26. Distefano, M., Ferlini, C., De Vincenzo, R., Gaggini, C., Mancuso, S., and Scambia, G. (2000). Antagonistic effect of the combination gemcitabine/topotecan in ovarian cancer cells. Oncol. Res. *12*, 355–359. https://doi.org/10.3727/096504001108747819.
27. Choi, S.I., Jeon, A.R., Kim, M.K., Lee, Y.S., Im, J.E., Koh, J.W., Han, S.S., Kong, S.Y., Yoon, K.A., Koh, Y.H., et al. (2019). Development of Patient-Derived Preclinical Platform for Metastatic Pancreatic Cancer: PDOX and a Subsequent Organoid Model System Using Percutaneous Biopsy Samples. Front. Oncol. *9*, 875. https://doi.org/10.3389/fonc.2019.00875.
28. Guichard, S., Hennebelle, I., Bugat, R., and Canal, P. (1998). Cellular interactions of 5-fluorouracil and the camptothecin analogue CPT-11 (irinotecan) in a human colorectal carcinoma cell line. Biochem. Pharmacol. *55*, 667–676. https://doi.org/10.1016/s0006-2952(97)00541-8.
29. NCCN (2022). NCCN Clinical Practice Guidelines in Oncology: Rectal Cancer
30. Zhu, J., Liu, A., Sun, X., Liu, L., Zhu, Y., Zhang, T., Jia, J., Tan, S., Wu, J., Wang, X., et al. (2020). Multicenter, Randomized, Phase III Trial of Neoadjuvant Chemoradiation With Capecitabine and Irinotecan Guided by UGT1A1 Status in Patients With Locally Advanced Rectal Cancer. J. Clin. Oncol. *38*, 4231–4239. https://doi.org/10.1200/jco.20.01932.
31. Lv, T., Shen, L., Xu, X., Yao, Y., Mu, P., Zhang, H., Wan, J., Wang, Y., Guan, R., Li, X., et al. (2023). Patient-derived tumor organoids predict responses to irinotecan-based neoadjuvant chemoradiotherapy in patients with locally advanced rectal cancer. Int. J. Cancer *152*, 524–535. https://doi.org/10.1002/ijc.34302.
32. DeVita, V.T., Jr., and Rosenberg, S.A. (2012). Two hundred years of cancer research. N. Engl. J. Med. *366*, 2207–2214. https://doi.org/10.1056/NEJMra1204479.
33. Ribas, A., and Wolchok, J.D. (2018). Cancer immunotherapy using checkpoint blockade. Science *359*, 1350–1355. https://doi.org/10.1126/science.aar4060.
34. Cancer Genome Atlas Network (2012). Comprehensive molecular characterization of human colon and rectal cancer. Nature *487*, 330–337. https://doi.org/10.1038/nature11252.
35. Demaria, S., and Formenti, S.C. (2012). Radiation as an immunological adjuvant: current evidence on dose and fractionation. Front. Oncol. *2*, 153. https://doi.org/10.3389/fonc.2012.00153.
36. Barker, H.E., Paget, J.T.E., Khan, A.A., and Harrington, K.J. (2015). The tumour microenvironment after radiotherapy: mechanisms of resistance and recurrence. Nat. Rev. Cancer *15*, 409–425. https://doi.org/10.1038/nrc3958.
37. Jeggo, P., and Lavin, M.F. (2009). Cellular radiosensitivity: how much better do we understand it? Int. J. Radiat. Biol. *85*, 1061–1081. https://doi.org/10.3109/09553000903261263.
38. Sebag-Montefiore, D., Adams, R., Gollins, S., Samuel, L.M., Glynne-Jones, R., Harte, R., West, N., Quirke, P., Myint, A.S., Bach, S.P., et al. (2020). ARISTOTLE: A phase III trial comparing concurrent capecitabine with capecitabine and irinotecan (Ir) chemoradiation as preoperative treatment for MRI-defined locally advanced rectal cancer (LARC). J. Clin. Oncol. *38*, 4101. https://doi.org/10.1200/JCO.2020.38.15_suppl.4101.
39. Greenhalgh, T.A., Dearman, C., and Sharma, R.A. (2016). Combination of Novel Agents with Radiotherapy to Treat Rectal Cancer. Clin. Oncol. *28*, 116–139. https://doi.org/10.1016/j.clon.2015.11.002.
40. Deng, Y., Chi, P., Lan, P., Wang, L., Chen, W., Cui, L., Chen, D., Cao, J., Wei, H., Peng, X., et al. (2019). Neoadjuvant Modified FOLFOX6 With or Without Radiation Versus Fluorouracil Plus Radiation for Locally Advanced Rectal Cancer: Final Results of the Chinese FOWARC Trial. J. Clin. Oncol. *37*, 3223–3233. https://doi.org/10.1200/jco.18.02309.
41. Sartorius, D., Blume, M.L., Fleischer, J.R., Ghadimi, M., Conradi, L.C., and De Oliveira, T. (2023). Implications of Rectal Cancer Radiotherapy on the Immune Microenvironment: Allies and Foes to Therapy Resistance and Patients' Outcome. Cancers (Basel) *15*, 5124. https://doi.org/10.3390/cancers15215124.
42. Pedrosa, L., Esposito, F., Thomson, T.M., and Maurel, J. (2019). The Tumor Microenvironment in Colorectal Cancer Therapy. Cancers (Basel) *11*, 1172. https://doi.org/10.3390/cancers11081172.
43. Schrag, D., Weiser, M.R., Goodman, K.A., Gonen, M., Hollywood, E., Cercek, A., Reidy-Lagunes, D.L., Gollub, M.J., Shia, J., Guillem, J.G., et al. (2014). Neoadjuvant chemotherapy without routine use of radiation therapy for patients with locally advanced rectal cancer: a pilot trial. J. Clin. Oncol. *32*, 513–518. https://doi.org/10.1200/jco.2013.51.7904.
44. Ryan, R., Gibbons, D., Hyland, J.M.P., Treanor, D., White, A., Mulcahy, H. E., O'Donoghue, D.P., Moriarty, M., Fennelly, D., and Sheahan, K. (2005). Pathological response following long-course neoadjuvant chemoradiotherapy for locally advanced rectal cancer. Histopathology *47*, 141–146. https://doi.org/10.1111/j.1365-2559.2005.02176.x.
45. Bando, H., Tsukada, Y., Inamori, K., Togashi, Y., Koyama, S., Kotani, D., Fukuoka, S., Yuki, S., Komatsu, Y., Homma, S., et al. (2022). Preoperative Chemoradiotherapy plus Nivolumab before Surgery in Patients with Microsatellite Stable and Microsatellite Instability-High Locally Advanced Rectal Cancer. Clin. Cancer Res. *28*, 1136–1146. https://doi.org/10.1158/1078-0432.Ccr-21-3213.
46. Ryu, H.S., Lee, J.L., Kim, C.W., Yoon, Y.S., Park, I.J., Lim, S.B., Yu, C.S., Kim, J.H., and Kim, J.C. (2022). Correlative Significance of Tumor Regression Grade and ypT Category in Patients Undergoing Preoperative Chemoradiotherapy for Locally Advanced Rectal Cancer. Clin. Colorectal Cancer *21*, 212–219. https://doi.org/10.1016/j.clcc.2022.02.001.
47. Zhang, C., Xu, L., Qin, Q., Liu, J., Tang, X., Jiang, N., Zhang, Z., Li, F., Cheng, H., Chen, J., and Sun, X. (2019). Good response to neoadjuvant chemoradiotherapy predicts good oncological outcome in locally advanced rectal cancer. Transl. Cancer Res. *8*, 150–159. https://doi.org/10.21037/tcr.2019.01.17.

STAR★METHODS

KEY RESOURCES TABLE

REAGENT or RESOURCE	SOURCE	IDENTIFIER
Chemicals, peptides, and recombinant proteins		
Advanced DMEM/F12	Gibco	Cat# 12634-010
R-spondin 1	Sino Biological	Cat# 11083-HNAS
Noggin	Sino Biological	Cat# 50688-M02H
R-spondin 1	EnaMab	Cat# ERSP1000
Noggin	EnaMab	Cat# ENGG0100
Wnt3a	EnaMab	Cat# EWNT0100
EGF	Sino Biological	Cat# 50482-MNCH
HEPES	Gibco	Cat# 15630080
Glutamax	Gibco	Cat# 35050061
Normocin	InvivoGen	Cat# ant-nr-1
Gentamicin/amphoteritin B	Gibco	Cat# R01510
N2 Supplement	Invitrogen	Cat# 17502-048
B27 Supplement	Invitrogen	Cat# 17504-044
N-Acetylcysteine	Sigma-aldrich	Cat# A9165
Niacinamide	Sigma-aldrich	Cat# N0636
A-83-01	Tocris	Cat# 2939
SB202190	Sigma-aldrich	Cat# S7067
Gastrin	Sigma-aldrich	Cat# G9145
Prostaglandin E2	Sigma-aldrich	Cat# P6532
Penicillin/streptomycin	Gibco	Cat# 15140-122
DMEM medium	Gibco	Cat# C1199500BT
Collagenase IV	Sigma-aldrich	Cat# C9407
Collagenase II	Solarbio	Cat# C8150
Hyaluronidase	Solarbio	Cat# h8030
Dispase type II	Sigma-aldrich	Cat# D4693
RHOK inhibitor ly27632	Sigma-aldrich	Cat# Y0503
Matrigel	Corning	Cat# 356231
Matrixtru	EnaMab	Cat# EXCT0805
Albumin Bovine	BBI Life Science	Cat# A600332-0100
TrypLETM Express	Gibco	Cat#12605-010
CELLBANKER^{TM} 2	ZENOAQ	Cat#170905
5-Fluorouracil	Selleck	Cat# S1209
Irinotecan	Selleck	Cat# S2217
Oxaliplatin	Selleck	Cat# S1224
Phosphate buffered saline	BasalMedia	Cat# B310KJ
Fetal bovine serum	Gibco	Cat# 10091148
Deposited data		
Figures S1–S4 and Tables S3–S10	Mendeley Database	DOI: https://doi.org/10.17632/z6mdxvzwky.1
Experimental models: Cell lines		
Human: rectal cancer organoids	This study	N/A

(*Continued on next page*)

Continued

REAGENT or RESOURCE	SOURCE	IDENTIFIER
Software and algorithms		
Image-Pro Plus 6.0	Media Cybernetics, Inc	http://www.mediacy.com/78-products/image-pro-plus
ImageJ software		https://imagej.nih.gov/ij/
SPSS 19.0	IBM	https://www.ibm.com/analytics/spss-statistics-software
R software	GNU project	https://www.r-project.org/
Other		
Phase III clinical trial: Trial of Capecitabine With or Without Irinotecan Driven by UGT1A1 (CinClare)		https://www.clinicaltrials.gov/ct2/show/NCT02605265?term=NCT02605265&rank=1

EXPERIMENTAL MODEL AND STUDY PARTICIPANT DETAILS

Patient information and human specimens

All rectal cancer tissue collections and associated experiments were reviewed and approved by the Institutional Review Boards of Fudan University Shanghai Cancer Center. Informed written consent was obtained from each patient before rectal biopsy. The biopsies were performed by experienced radiation oncologists following the procedural guidelines outlined in our previous study. All patients involved in this study were LARC patients enrolled in a CinClare trial[30](a multicenter phase 3 clinical trial, NCT02605265) and LARC patients treated with the same chemoradiotherapy regimens in the Department of Radiation Oncology, Fudan University Shanghai Cancer Center. In the CinClare trial, eligible patients were randomly allocated to the control group: pelvic radiation of 50 Gy/25 fractions with concurrent capecitabine; or the experimental group: radiation with capecitabine combined with weekly irinotecan. The clinical data of corresponding patients including patient baseline characteristics, MRI imaging materials, and pathological tumor regression grade (TRG) were independently collected by radiation oncologists from the medical records system. The studies were conducted in accordance with recognized ethical guidelines (Declaration of Helsinki).

Rectal cancer organoids culture medium

Rectal cancer organoids were cultured in Advanced DMEM/F12 medium supplemented with a combination of various growth factors and inhibitors, including R-spondin 1, Noggin, EGF, HEPES, Glutamax, Normocin, Gentamicin/amphotericin B, N2, B27, n-Acetylcysteine, Niacinamide, Alk 4/5/7 inhibitor, p38 inhibitor, Gastrin, and Prostaglandin E2, with or without Wnt3a. Specific details of the culture conditions are provided in Table S12.

METHOD DETAILS

Tumor cell isolation and organoid culture

The rectal cancer (RC) tissues were immersed in cold PBS containing penicillin/streptomycin and transported to the laboratory. The subsequent procedures for tumor cell isolation and organoid culture were consistent with those described in our previous study.[4] To improve the success rate and reproducibility of organoid establishment, we optimized several key steps in our protocol. First, tumor specimen quality was ensured by having experienced endoscopists collect biopsy tissues, which were immediately stored in tissue preservation solution and transported at 4°C to the laboratory within six hours of collection. Second, contamination control was enhanced by supplementing both the tissue preservation medium and the PBS washing buffer with antibiotics. Tissues were washed on ice at least five times, each for five minutes, which effectively eliminated microbial contamination. Third, to ensure the viability of tumor epithelial clusters, tissue digestion time was strictly controlled to 15–20 min, with intermittent shaking (30 s every 5 min), avoiding continuous enzymatic digestion. After digestion, pipetting was minimized to prevent mechanical damage, and digestion efficiency was modulated by adjusting enzyme volume rather than extending processing time. All procedures were completed within 90 min. Fourth, we used two parallel culture media systems—one based on commercialized growth factor cocktails and the other using a customized homemade medium—to increase the overall success rate. Finally, the first organoid passaging was conducted 9–12 days after initial seeding, as earlier passaging (e.g., within six days) was found to compromise viability and growth. These protocol refinements significantly enhanced the efficiency and consistency of rectal cancer organoid establishment.

Drug/irradiation screening

To assess drug/irradiation sensitivity, tumor organoids were seeded at 100–200 organoids per well in a 48-well plate. The complete process of drug/irradiation screening lasted for 24 days, including 6 days of culture in the drug-containing medium (10 μM 5-FU

(Selleck, S1209) or 10 μM irinotecan (Selleck, S2217)), followed by 18 days of culture in fresh medium. The medium was refreshed every three days. Irradiation was administered at a dose of 8 Gy on the day the drug screening began.

The concentrations of 5-FU/irinotecan and the radiation dosage used in this study were in accordance with those outlined in our previous study.[4] To model clinical neoadjuvant chemoradiotherapy *in vitro*, we treated patient-derived rectal cancer organoids with irradiation and chemotherapeutic agents at fixed doses. In clinical practice, patients with locally advanced rectal cancer (cT3–4N0–2M0) typically receive a total of 50 Gy radiation delivered in 25 fractions. To identify an appropriate single-dose irradiation scheme for organoid assays, we performed exploratory experiments testing a range of irradiation doses. Among the doses evaluated, 8 Gy was selected based on its ability to elicit differential responses across organoid lines, thereby enabling response stratification. This dose captured inter-patient heterogeneity and was consistent with the diverse responses observed in clinical NACR outcomes. Chemotherapy agents, including 5-Fu and CPT-11, were applied at concentrations of 10 μM, which were determined through similar exploratory dose optimization studies. These concentrations yielded discriminative response profiles among organoid lines while maintaining cell viability suitable for downstream analyses. The rationale and supporting data for the selected doses of irradiation and chemotherapeutics were described in our previous work,[4] particularly in Figures S2 and S3, and were additionally informed by data from other organoid studies.[11]

Organoid sizes were recorded using (ZEISS, Vert. A1) every three days. The change in organoid size was determined using Image-Pro Plus 6.0 (Media Cybernetics, Inc) and ImageJ (National Institute of Health, USA). The sizes of viable organoids were quantified using Image-Pro Plus 6.0 (Media Cybernetics, Inc.).

Criteria for defining synergism and antagonism

In evaluating potential interactions between chemotherapeutic agents and irradiation, we considered established synergy models such as Bliss independence, Loewe additivity, and the ZIP (Zero Interaction Potency) model. These approaches are widely used in pharmacological studies to assess drug–drug interactions based on dose–response matrices. However, their application requires a series of viability measurements across multiple concentrations of each agent and their combinations, enabling mathematical modeling of independent and additive effects. In contrast, our experimental design involved fixed-dose treatments evaluated over an extended time-course (day 0 to day 24), focusing on dynamic response patterns rather than dose–response relationships. As such, traditional synergy quantification methods were not applicable. Instead, we employed a binary response framework using a predefined viability ratio cutoff (24-day/day-0 area ratio of 34.87%) to classify organoid responses. Under this approach, synergy was conservatively defined as resistance to both single-agent treatments and sensitivity to the combination. Antagonism was defined when sensitivity to at least one monotherapy was lost upon combination treatment. This classification was designed to provide an interpretable, robust estimate of interactive effects within the constraints of our data structure.

Clinical response evaluation

The clinical complete response (cCR) status was determined based on comprehensive evaluations including the digital rectal examination (DRE), endoscopic examination, and rectal MRI found no evidence of disease recurrence for a minimum duration of one year, independently conducted by two experienced oncologists. For patients who reached cCR, the Wait and Watch strategy including close monitoring by endoscopy, DRE, and pelvic MRI was administrated.

Several tumor regression grade (TRG) systems have been developed to assess primary tumor response to neoadjuvant chemoradiotherapy, including the Mandard five-tier, Dowrak/Rödel five-tier, AJCC four-tier, MSKCC three-tier, Mandard three-tier, and Dowrak/Rödel three-tier systems. Among these, the AJCC Staging Manual (7th edition) system has been validated as the most accurate and was adopted as the standard at Shanghai Cancer Center, Fudan University.[16] The AJCC four-tier TRG system, originally derived from the Ryan classification, has been adopted as the standard pathological grading system for evaluating response to neoadjuvant therapy in rectal cancer.[43,44] For patients who received surgery after neoadjuvant chemoradiotherapy, pTRGs were evaluated by experienced pathologists according to The American Joint Committee on Cancer (AJCC) Staging Manual (7th edition) system. pTRG 0 was defined as the absence of viable cancer cells; the presence of a solitary or minor residual cancer cell was categorized as pTRG 1; pTRG 2 indicated the existence of residual cancer cells more than fibrosis; minimal or no tumor cells killed were classified as TRG3.

The evaluation of cCR and the assessment of pTRG after surgery served as the established standard to evaluate the patient's response to NACR. In this study, patients who reached cCR, pTRG = 0 or 1 were sensitive to NACR, whereas patients who had pTRG of 2–3 were resistant to NACR. TRG2 corresponds to a partial response in the original AJCC/Ryan classification. However, in our study, patients were dichotomized into "sensitive" and "resistant" groups in order to facilitate comparative analysis between responders and non-responders to neoadjuvant chemoradiotherapy. This binary grouping was based not only on histological definitions but also on clinical outcomes. Previous studies have shown that patients with TRG 0–1 have significantly better long-term outcomes than those with TRG 2–3. For instance, the 5-year recurrence-free survival (RFS) rates were 98% (TRG 0), 90% (TRG 1), 73% (TRG 2), and 68% (TRG 3).[16] Therefore, despite TRG 2 representing a partial histologic response, its survival profile aligns more closely with TRG 3 than with TRG 0–1. This clinically oriented rationale guided our classification. This classification strategy has also been adopted in prior studies.[45–47]

QUANTIFICATION AND STATISTICAL ANALYSIS

Patient-derived rectal cancer organoids were classified as either sensitive or resistant to treatment based on changes in organoid size following exposure to irradiation and chemotherapeutic agents (5-FU and CPT-11). Organoid response was quantified as the ratio of size on day 24 relative to day 0. The cutoff value for this ratio was determined by maximizing Youden's J index—equivalent to maximizing the sum of sensitivity and specificity—across all points of the receiver operating characteristic (ROC) curve. In our previous study, which used monotherapy conditions (irradiation, 5-FU, or CPT-11 alone), the lowest ratio among the three treatment arms for each sample was used as a univariate predictor.[4] In the current study, we applied the same methodology using data from the combined treatment condition (irradiation + chemotherapy). To evaluate the predictive performance of organoid response for clinical outcomes, we calculated standard diagnostic metrics, including area under the ROC curve (AUC), sensitivity, specificity, and overall accuracy. Agreement between organoid-based classification and clinical response was assessed using Cohen's Kappa coefficient, a measure of inter-rater reliability. To address potential overfitting, we implemented 1,000 bootstrap simulations to estimate the distribution of out-of-bag (OOB) diagnostic metrics. The final cutoff value based on Youden's index was selected from these bootstrap samples, and all performance measures were reported as mean values with 95% confidence intervals. For comparisons of categorical variables, chi-square tests were used. A p-value less than 0.05 was considered statistically significant. All statistical analyses were performed using R software (version 3.5.1).

Cell Reports Medicine, Volume *6*

Supplemental information

Comprehensive dissection of rectal

cancer organoids in responses to chemoradiation

Xiaoya Xu, Tao Lv, Ye Yao, Juefeng Wan, Lijun Shen, Fan Xia, Xiaoxue Gao, Yuanchuang Li, Guoxiang Fu, Yun Deng, Mengxue Pan, Qiang Guo, Xinxin Rao, Peiyuan Tang, Xiaomeng Li, Yi Zhou, Liping Liang, Yaqi Wang, Jing Zhang, Hui Zhang, Guichao Li, Min Chen, Junjie Peng, Sanjun Cai, Jianjun Gao, Guoqiang Hua, and Zhen Zhang

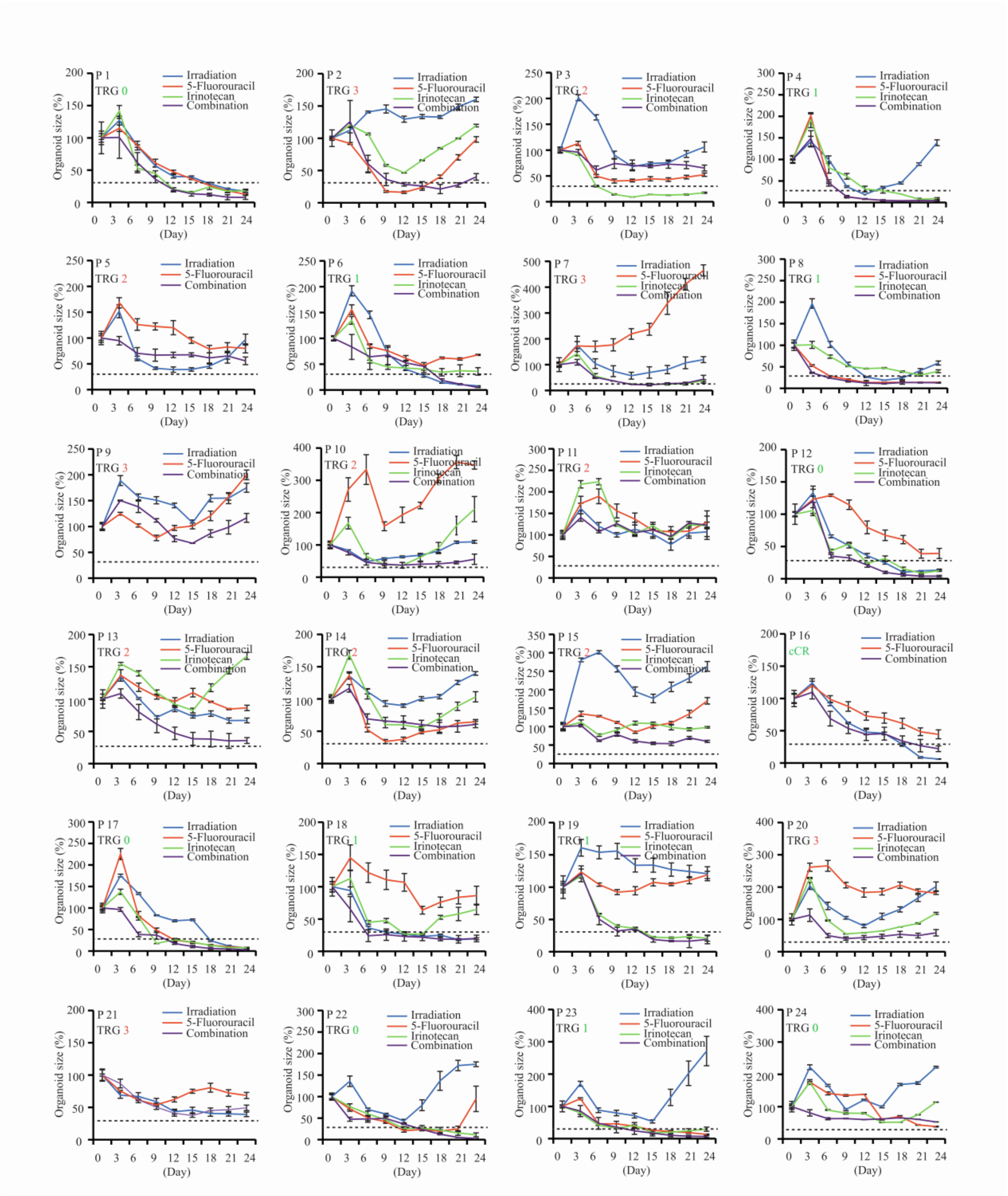

Supplementary Figure 1. The detailed data of RCO lines (P1-P24) size change after single treatments and combined chemoradiation. Organoids size data shown were means from three independent experiments (n = 12). Red curves, patients with a poor clinical response (TRG 2 or 3); green curves, patients with a good clinical response (TRG 0 or 1 or cCR). Dotted lines indicated cutoff of organoid size change (34.87%), Related to Figure 1 and Figure 2.

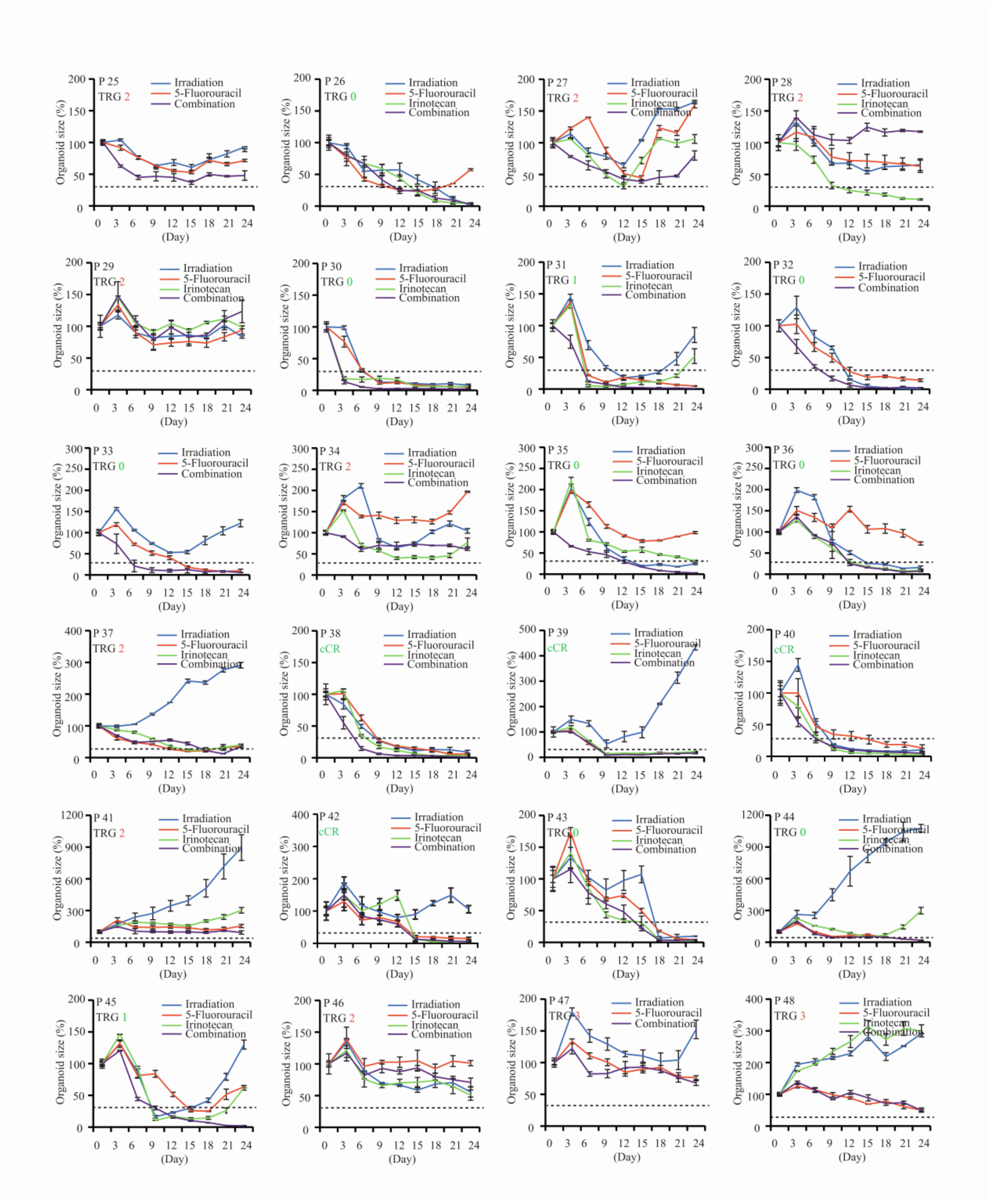

Supplementary Figure 2. The detailed data of RCO lines (P25-P48) size change after single treatments and combined chemoradiation. Organoids size data shown were means from three independent experiments (n = 12). Red curves, patients with a poor clinical response (TRG 2 or 3); green curves, patients with a good clinical response (TRG 0 or 1 or cCR). Dotted lines indicated cutoff of organoid size change (34.87%), Related to Figure 1 and Figure 2.

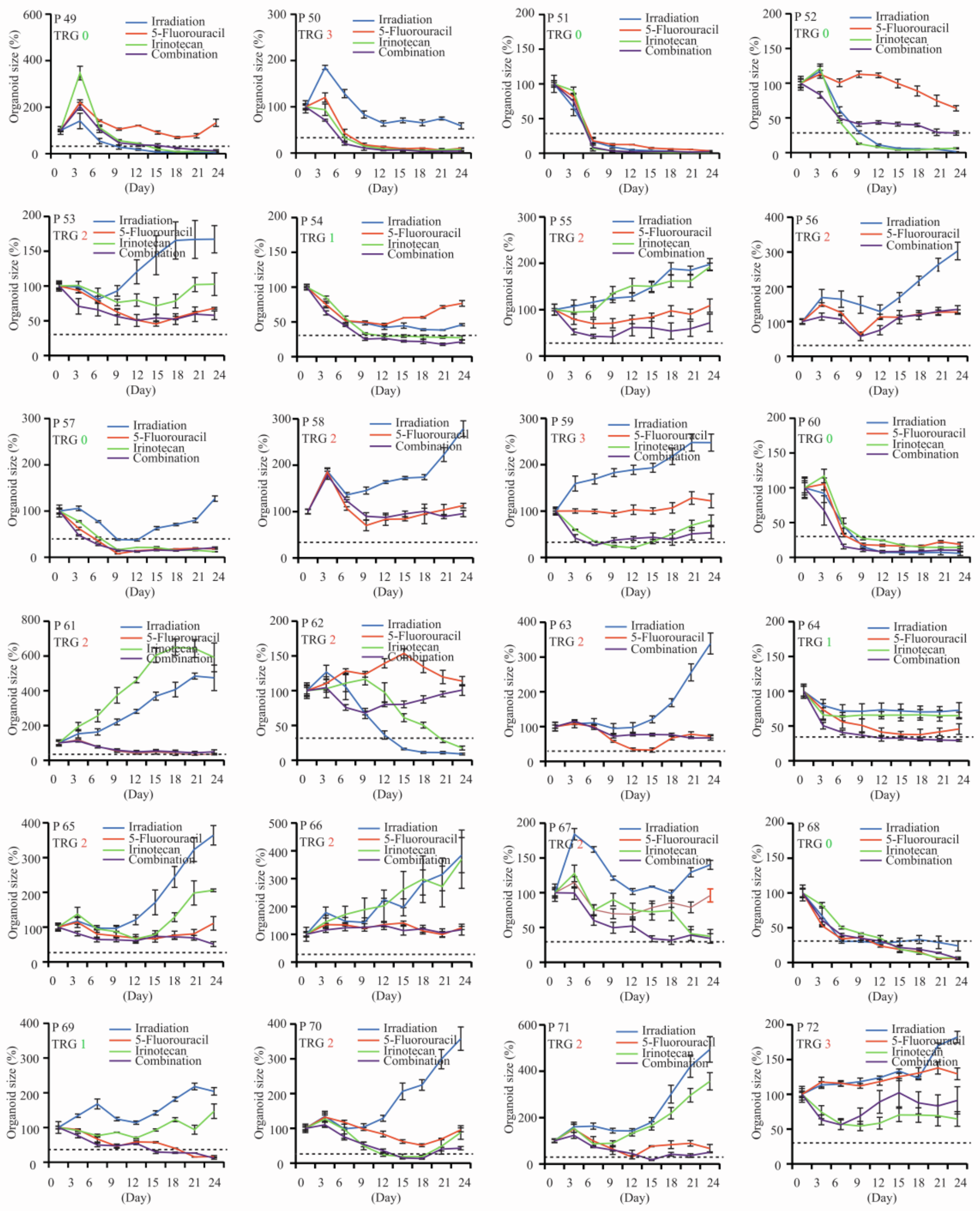

Supplementary Figure 3. The detailed data of RCO lines (P49-P72) size change after single treatments and combined chemoradiation. Organoids size data shown were means from three independent experiments (n = 12). Red curves, patients with a poor clinical response (TRG 2 or 3); green curves, patients with a good clinical response (TRG 0 or 1 or cCR). Dotted lines indicated cutoff of organoid size change (34.87%), Related to Figure 1 and Figure 2.

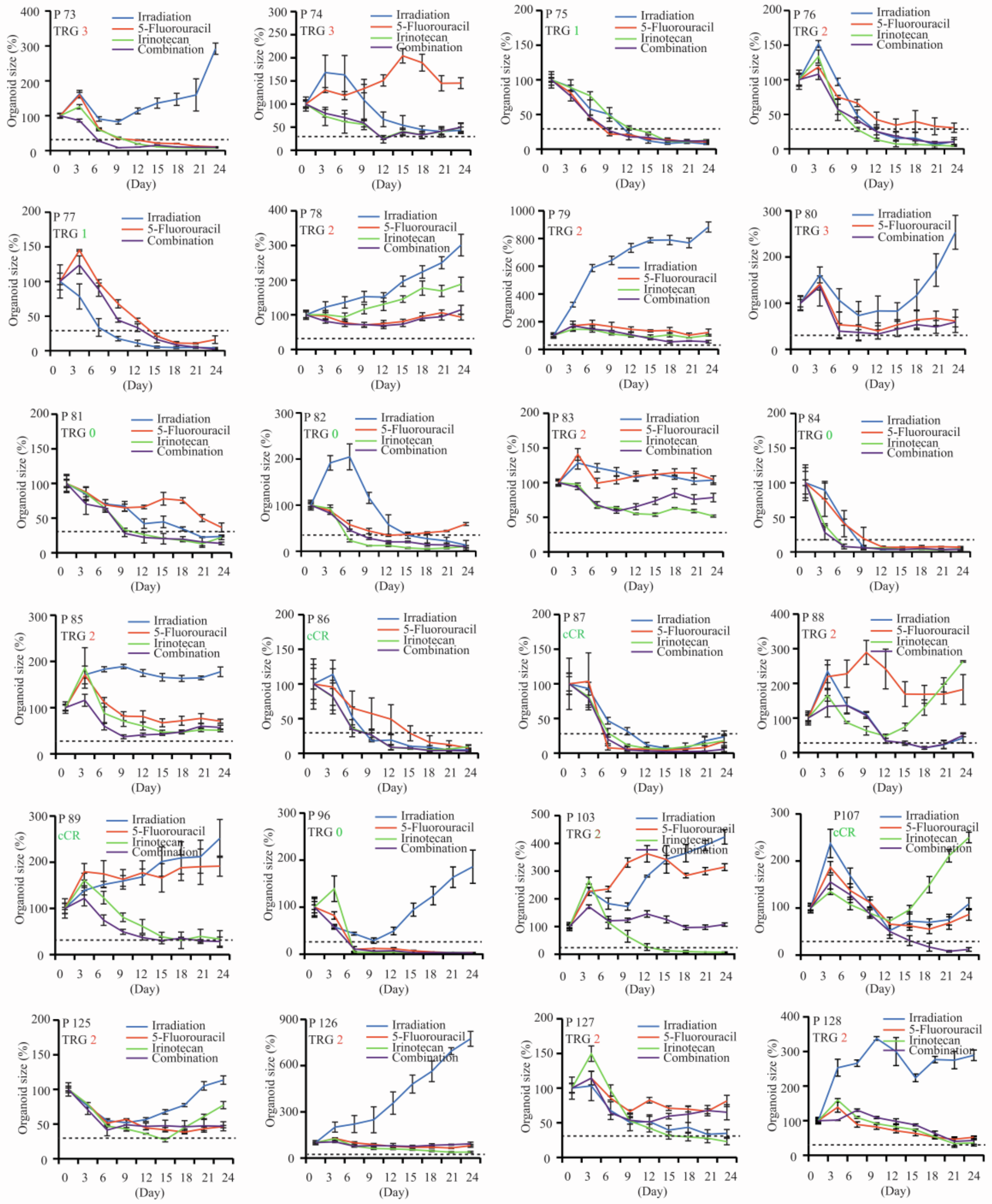

Supplementary Figure 4. The detailed data of RCO lines (P73-P89, P96, P103, P107, P125-P128) size change after single treatments and combined chemoradiation. Organoids size data shown were means from three independent experiments (n = 12). Red curves, patients with a poor clinical response (TRG 2 or 3); green curves, patients with a good clinical response (TRG 0 or 1 or cCR). Dotted lines indicated cutoff of organoid size change (34.87%), Related to Figure 1 and Figure 2.

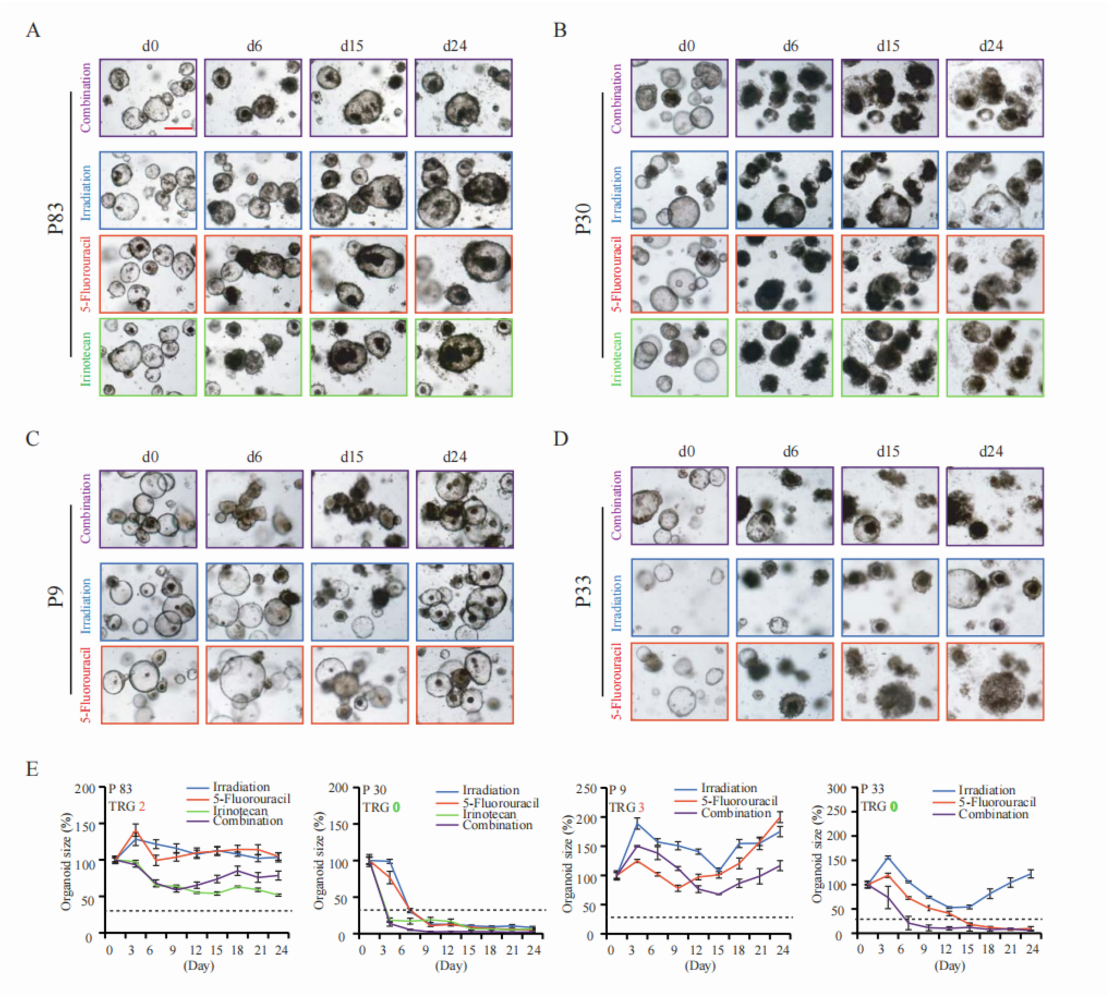

Supplementary Figure 5。Related to Figure 4:

(A,C) Representative bright-filed images of two selected organoid lines (O83 and O9) which were resistant to single treatments of irradiation, 5-Fu and CPT-11 and combined chemoradiation at day0, 6, 15 and 24 after treatments. Scale bars, 200 µm. (B,D) Representative bright-filed images of two selected organoid lines (O30 and O33) which were sensitive to single treatment and combined chemoradiation at day0, 6, 15 and 24 after treatments. (E) Size change curves of organoids after single treatments and combined chemoradiation in 4 selected cases (O83, O30, O9, O33). Organoids size data shown were means±SEM from three independent experiments (n = 12). Red curves, patients with a poor clinical response (TRG 2 or 3); green curves, patients with a good clinical response (TRG 0 or 1 or cCR). Dotted lines indicated cutoff of organoid size change (34.87%).

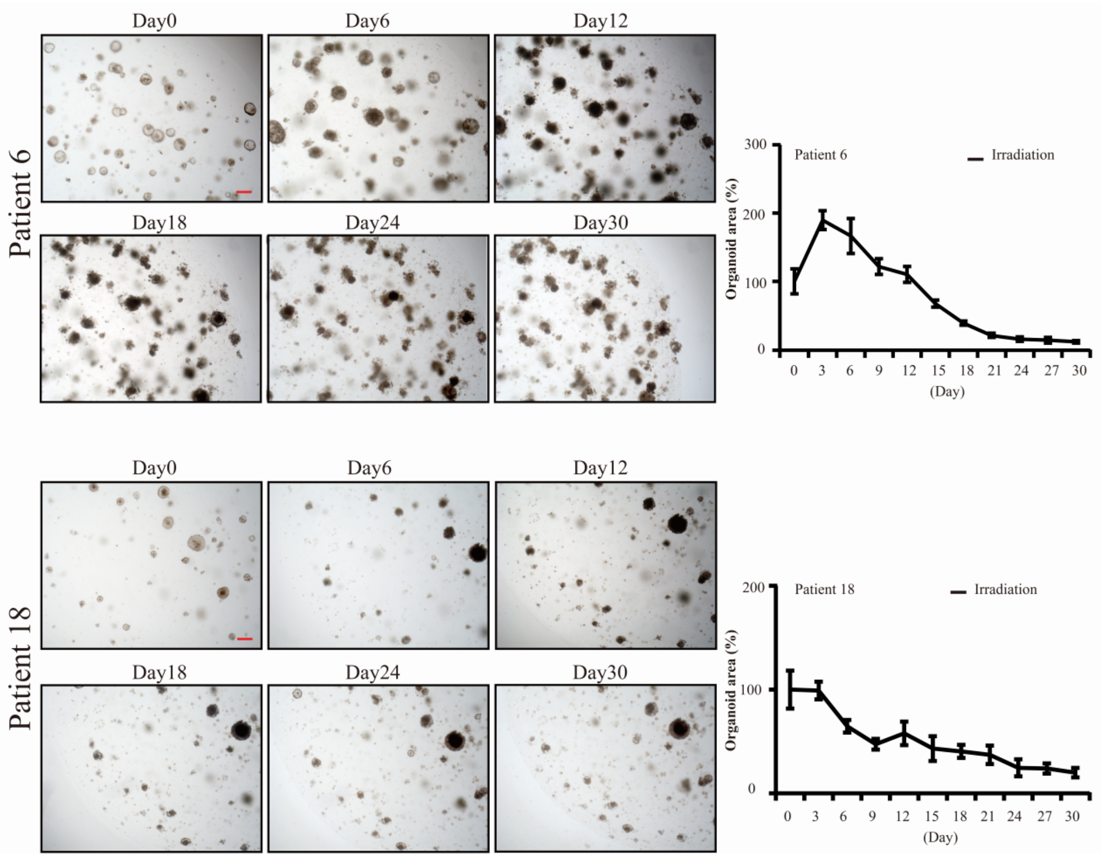

Supplementary Figure 6。 Related to Figure 3.

Representative bright-field images and size change curves of organoids after irradiation in 2 selected cases from day 0 to day 30. Viable organoids exhibited intact and complete structures, whereas non-viable organoids displayed disrupted and fragmented morphology. Organoids size data shown were means±SEM from three independent experiments (n = 12). Scale bar, 200 μm.

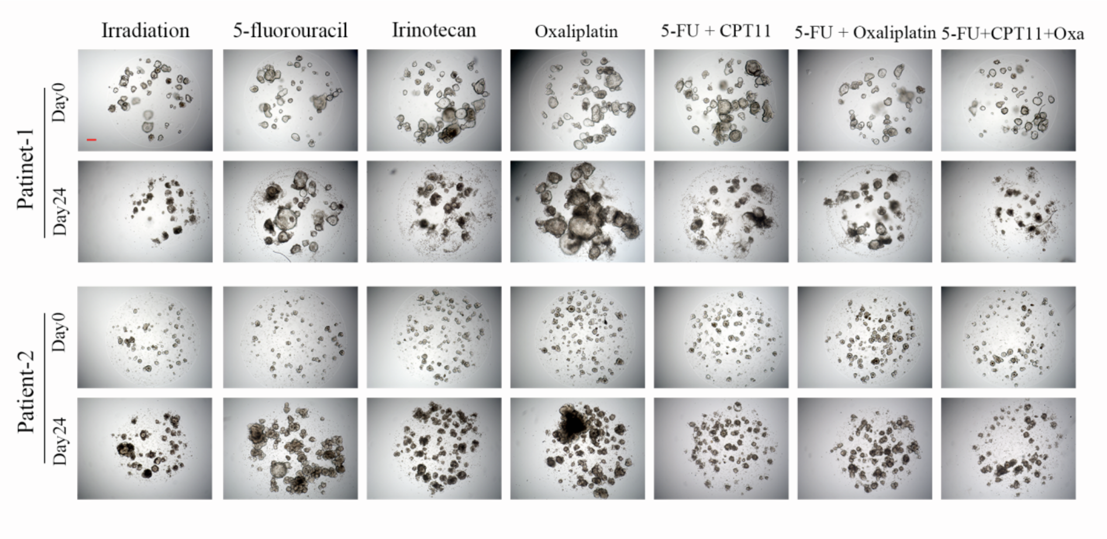

Supplementary Figure 7. Related to Figure 1 and Figure 4.

Representative bright-field images of two RCO lines at day 0 and day 24 after treatment with single or combined regimens, including FOLFOX, FOLFIRI, and FOLFOXIRI. Viable organoids exhibited intact and complete structures, whereas non-viable organoids displayed disrupted and fragmented morphology. Scale bar, 200 μm.

Supplementary Table 1. Clinical data of 128 patients, Related to Figure 1 and Figure2.

Number	Gender	Age	T	N	Histopathology	Treatments	TRG
Patient-1	male	54	T3	N2b	Adenocarcinoma	radiation/irinotecan/Capecitabine	0
Patient-2	female	34	T3b	N2b	Adenocarcinoma	radiation/irinotecan/Capecitabine	3
Patient-3	female	62	T3b	N2b	Adenocarcinoma	radiation/irinotecan/Capecitabine	2
Patient-4	male	50	T3a	N1c	Adenocarcinoma	radiation/irinotecan/Capecitabine	1
Patient-5	male	49	T4a	N2b	Adenocarcinoma	radiation/Capecitabine	2
Patient-6	female	56	T3a	N1b	Adenocarcinoma	radiation/irinotecan/Capecitabine	1
Patient-7	male	30	T3b	N2b	Adenocarcinoma	radiation/irinotecan/Capecitabine	3
Patient-8	female	25	T3a	N1b	Adenocarcinoma	radiation/irinotecan/Capecitabine	1
Patient-9	male	33	T3b	N2	Adenocarcinoma	radiation/Capecitabine	3
Patient-10	male	49	T3a	N2a	Adenocarcinoma	radiation/irinotecan/Capecitabine	2
Patient-11	female	69	T3c	N2b	Adenocarcinoma	radiation/irinotecan/Capecitabine	2
Patient-12	male	46	T3a	N1b	Adenocarcinoma	radiation/irinotecan/Capecitabine	0
Patient-13	male	55	T3b	N2a	Adenocarcinoma	radiation/irinotecan/Capecitabine	2
Patient-14	male	53	T3a	N2a	Adenocarcinoma	radiation/irinotecan/Capecitabine	2
Patient-15	male	64	T4b	N2a	Adenocarcinoma	radiation/irinotecan/Capecitabine	2
Patient-16	male	52	T3a	N2b	Adenocarcinoma	radiation/Capecitabine	ccr
Patient-17	male	55	T3a	N2a	Adenocarcinoma	radiation/irinotecan/Capecitabine	0
Patient-18	male	61	T3a	N1a	Adenocarcinoma	radiation/irinotecan/Capecitabine	1
Patient-19	female	38	T4b	N2b	Adenocarcinoma	radiation/irinotecan/Capecitabine	1
Patient-20	female	43	T3b	N2b	Adenocarcinoma	radiation/irinotecan/Capecitabine	3
Patient-21	male	64	T3b	N1b	Adenocarcinoma	radiation/Capecitabine	3
Patient-22	male	56	T3	N2b	Adenocarcinoma	radiation/Capecitabine	0
Patient-23	male	54	T3	N1	Adenocarcinoma	radiation/irinotecan/Capecitabine	1
Patient-24	male	56	T2	N2a	Adenocarcinoma	radiation/Capecitabine	0
Patient-25	male	60	T3a	N1b	Adenocarcinoma	radiation/Capecitabine	2
Patient-26	male	51	T3b	N2a	Adenocarcinoma	radiation/irinotecan/Capecitabine	0
Patient-27	male	71	T3b	N1b	Adenocarcinoma	radiation/irinotecan/Capecitabine	2
Patient-28	male	53	T3c	N2b	Adenocarcinoma	radiation/irinotecan/Capecitabine	2
Patient-29	female	63	T3	N2	Adenocarcinoma	radiation/irinotecan/Capecitabine	2
Patient-30	male	52	T3	N1	Adenocarcinoma	radiation/irinotecan/Capecitabine	0
Patient-31	male	53	T3c	N2b	Adenocarcinoma	radiation/irinotecan/Capecitabine	1
Patient-32	female	38	T4b	N2b	Adenocarcinoma	radiation/Capecitabine	0
Patient-33	male	44	T3b	N2a	Adenocarcinoma	radiation/Capecitabine	0
Patient-34	male	35	T3c	N2a	Adenocarcinoma	radiation/irinotecan/Capecitabine	2
Patient-35	male	69	T3a	N2a	Adenocarcinoma	radiation/irinotecan/Capecitabine	0
Patient-36	male	58	T3b	N1b	Adenocarcinoma	radiation/irinotecan/Capecitabine	0
Patient-37	male	65	T3a	N2a	Adenocarcinoma	radiation/irinotecan/Capecitabine	2
Patient-38	male	53	T3a	N2a	Adenocarcinoma	radiation/irinotecan/Capecitabine	ccr
Patient-39	male	39	T2	N1b	Adenocarcinoma	radiation/irinotecan/Capecitabine	ccr
Patient-40	male	36	T3a	N1b	Adenocarcinoma	radiation/irinotecan/Capecitabine	ccr
Patient-41	male	32	T3	N+	Adenocarcinoma	radiation/irinotecan/Capecitabine	2
Patient-42	male	42	T3a	N1a	Adenocarcinoma	radiation/irinotecan/Capecitabine	ccr

Patient-43	male	57	T3a	N2a	Adenocarcinoma	radiation/irinotecan/Capecitabine	0
Patient-44	male	73	T3b	N2b	Adenocarcinoma	radiation/irinotecan/Capecitabine	0
Patient-45	female	53	T3b	N1b	Adenocarcinoma	radiation/irinotecan/Capecitabine	1
Patient-46	male	58	T4	N2a	Adenocarcinoma	radiation/irinotecan/Capecitabine	2
Patient-47	female	67	T2	N0	Adenocarcinoma	radiation/Capecitabine	3
Patient-48	male	53	T3	N+	Adenocarcinoma	radiation/irinotecan/Capecitabine	3
Patient-49	female	52	T3a	N0	Adenocarcinoma	radiation/irinotecan/Capecitabine	0
Patient-50	female	54	T3b	N2	Adenocarcinoma	radiation/irinotecan/Capecitabine	3
Patient-51	male	57	T3b	N2a	Adenocarcinoma	radiation/irinotecan/Capecitabine	0
Patient-52	male	38	T3a	N1a	Adenocarcinoma	radiation/irinotecan/Capecitabine	0
Patient-53	male	61	T3a	N1b	Adenocarcinoma	radiation/irinotecan/Capecitabine	2
Patient-54	male	58	T3a	N2a	Adenocarcinoma	radiation/irinotecan/Capecitabine	1
Patient-55	male	59	T3b	N1	Adenocarcinoma	radiation/irinotecan/Capecitabine	2
Patient-56	male	41	T3c	N2b	Adenocarcinoma	radiation/Capecitabine	2
Patient-57	male	63	T3	N1	Adenocarcinoma	radiation/irinotecan/Capecitabine	0
Patient-58	male	52	T4a	N2b	Adenocarcinoma	radiation/Capecitabine	2
Patient-59	female	30	T4b	N2a	Adenocarcinoma	radiation/irinotecan/Capecitabine	3
Patient-60	female	31	T2	N0	Adenocarcinoma	radiation/irinotecan/Capecitabine	0
Patient-61	male	43	T4a	N+	Adenocarcinoma	radiation/irinotecan/Capecitabine	2
Patient-62	male	65	T3b	N1b	Adenocarcinoma	radiation/irinotecan/Capecitabine	2
Patient-63	male	60	T3b	N1b	Adenocarcinoma	radiation/Capecitabine	2
Patient-64	female	56	T3b	N2a	Adenocarcinoma	radiation/irinotecan/Capecitabine	1
Patient-65	male	56	T3	N1	Adenocarcinoma	radiation/irinotecan/Capecitabine	2
Patient-66	male	65	T3a	N2a	Adenocarcinoma	radiation/irinotecan/Capecitabine	2
Patient-67	female	58	T3C	N2a	Adenocarcinoma	radiation/irinotecan/Capecitabine	2
Patient-68	female	56	T2	N1a	Adenocarcinoma	radiation/irinotecan/Capecitabine	0
Patient-69	male	56	T3a	N2b	Adenocarcinoma	radiation/irinotecan/Capecitabine	1
Patient-70	male	63	T3a	N2a	Adenocarcinoma	radiation/irinotecan/Capecitabine	2
Patient-71	male	61	T4	N2a	Adenocarcinoma	radiation/irinotecan/Capecitabine	2
Patient-72	male	60	T3	Nx	Adenocarcinoma	radiation/irinotecan/Capecitabine	3
Patient-73	male	64	T3a	N1a	Adenocarcinoma	radiation/irinotecan/Capecitabine	3
Patient-74	male	46	T3b	N1b	Adenocarcinoma	radiation/irinotecan/Capecitabine	2
Patient-75	female	42	T3b	N1b	Adenocarcinoma	radiation/irinotecan/Capecitabine	1
Patient-76	male	44	T2	N1	Adenocarcinoma	radiation/irinotecan/Capecitabine	2
Patient-77	male	51	T3	N1	Adenocarcinoma	radiation/Capecitabine	1
Patient-78	male	47	T3a	N2a	Adenocarcinoma	radiation/irinotecan/Capecitabine	2
Patient-79	female	57	T3	N2	Adenocarcinoma	radiation/irinotecan/Capecitabine	2
Patient-80	male	47	T4a	N2b	Adenocarcinoma	radiation/Capecitabine	3
Patient-81	female	44	T3	N2	Adenocarcinoma	radiation/irinotecan/Capecitabine	0
Patient-82	male	50	T2	N0	Adenocarcinoma	radiation/irinotecan/Capecitabine	0
Patient-83	female	66	T3a	N0	Adenocarcinoma	radiation/Capecitabine	2
Patient-84	male	58	T2	N1b	Adenocarcinoma	radiation/irinotecan/Capecitabine	0
Patient-85	male	54	T3b	N2a	Adenocarcinoma	radiation/irinotecan/Capecitabine	2
Patient-86	male	57	T4	N2	Adenocarcinoma	radiation/Capecitabine	ccr

Patient-87	male	62	T4b	N2b	Adenocarcinoma	radiation/irinotecan/Capecitabine	ccr
Patient-88	female	83	T3a	N2b	Adenocarcinoma	radiation/Capecitabine	2
Patient-89	male	48	T2	N1	Adenocarcinoma	radiation/irinotecan/Capecitabine	ccr
Patient-90	male	64	T3b	N0	Adenocarcinoma	radiation/irinotecan/Capecitabine	2
Patient-91	female	48	T3a	N1b	Adenocarcinoma	radiation/irinotecan/Capecitabine	3
Patient-92	male	72	T2	N0	Adenocarcinoma	radiation/irinotecan/Capecitabine	2
Patient-93	male	72	T3	N+	Adenocarcinoma	radiation/Capecitabine	2
Patient-94	female	59	T3a	N0	Adenocarcinoma	radiation/irinotecan/Capecitabine	3
Patient-95	male	66	T3b	N2	Adenocarcinoma	radiation/irinotecan/Capecitabine	ccr
Patient-96	male	55	T3b	N1a	Adenocarcinoma	radiation/irinotecan/Capecitabine	0
Patient-97	male	55	T3	N3	Adenocarcinoma	radiation/irinotecan/Capecitabine	1
Patient-98	female	66	T4	N2	Adenocarcinoma	radiation/irinotecan/Capecitabine	0
Patient-99	female	33	T3a	N2b	Adenocarcinoma	radiation/irinotecan/Capecitabine	0
Patient-100	female	53	T3a	N0	Adenocarcinoma	radiation/irinotecan/Capecitabine	0
Patient-101	male	68	T3	N1	Adenocarcinoma	radiation/irinotecan/Capecitabine	0
Patient-102	female	69	T2	N0	Adenocarcinoma	radiation/irinotecan/Capecitabine	ccr
Patient-103	male	44	T4b	N1a	Adenocarcinoma	radiation/irinotecan/Capecitabine	2
Patient-104	male	58	T3	N2	Adenocarcinoma	radiation/irinotecan/Capecitabine	2
Patient-105	female	37	T2	N0	Adenocarcinoma	radiation/irinotecan/Capecitabine	ccr
Patient-106	female	65	T4	N2	Adenocarcinoma	radiation/irinotecan/Capecitabine	2
Patient-107	female	49	T2	N1a	Adenocarcinoma	radiation/irinotecan/Capecitabine	ccr
Patient-108	female	56	T3	N0	Adenocarcinoma	radiation/irinotecan/Capecitabine	ccr
Patient-109	male	38	T3	N1	Adenocarcinoma	irinotecan/Capecitabine	2
Patient-110	female	64	T3b	N2a	Adenocarcinoma	radiation/irinotecan/Capecitabine	2
Patient-111	female	48	T3a	N2b	Adenocarcinoma	radiation/irinotecan/Capecitabine	2
Patient-112	female	34	T4b	N2a	Adenocarcinoma	radiation/irinotecan/Capecitabine	2
Patient-113	male	61	T3C	N2a	Adenocarcinoma	radiation/irinotecan/Capecitabine	2
Patient-114	female	48	T3a	N1b	Adenocarcinoma	radiation/irinotecan/Capecitabine	1
Patient-115	female	55	T3C	N2b	Adenocarcinoma	radiation/Capecitabine	2
Patient-116	male	60	T3a	N1b	Adenocarcinoma	radiation/irinotecan/Capecitabine	2
Patient-117	male	70	T3	N0	Adenocarcinoma	radiation/Capecitabine	2
Patient-118	male	53	T3	N+	Adenocarcinoma	radiation/irinotecan/Capecitabine	3
Patient-119	male	40	T3a	N2a	Adenocarcinoma	radiation/irinotecan/Capecitabine	1
Patient-120	male	55	T3a	N1b	Adenocarcinoma	radiation/Capecitabine	0
Patient-121	male	62	T3a	N1b	Adenocarcinoma	radiation/irinotecan/Capecitabine	2
Patient-122	male	50	T3b	N2a	Adenocarcinoma	radiation/Capecitabine	2
Patient-123	male	65	T3a	N1b	Adenocarcinoma	radiation/irinotecan/Capecitabine	2
Patient-124	female	66	T4b	N1a	Adenocarcinoma	radiation/irinotecan/Capecitabine	0
Patient-125	female	63	T3a	N2a	Adenocarcinoma	radiation/irinotecan/Capecitabine	3
Patient-126	male	66	T2	N+	Adenocarcinoma	radiation/irinotecan/Capecitabine	2
Patient-127	male	49	T3a	N2a	Adenocarcinoma	radiation/irinotecan/Capecitabine	2
Patient-128	male	52	T4a	N2b	Adenocarcinoma	radiation/irinotecan/Capecitabine	2

Supplementary Table 2. Ratios of RCO size change on day24 to day0 after single treatments and combined chemoradiation, Related to Figure 1 and Figure 2.

Patients number	Irradiation	5-fluorouracil 6-(5-Fu)	Irinotecan (CPT-11)	Combined treatments
**Patient1**	0.1732	0.1245	0.1796	0.0779
**Patient2**	1.602	0.9826	1.1959	0.4014
**Patient3**	1.0596	0.5233	0.1737	0.6521
**Patient4**	1.3869	0.0449	0.0947	0.0426
**Patient5**	0.9694	0.7991	0.7702	0.5579
**Patient6**	0.082	0.6799	0.3706	0.0892
**Patient7**	1.1961	4.6461	0.3839	0.4358
**Patient8**	0.5862	0.1408	0.3961	0.1292
**Patient9**	1.7505	1.9983	1.7603	1.1668
**Patient10**	1.0941	3.4739	2.1068	0.55627
**Patient11**	1.0694	1.2971	1.2187	1.2265
**Patient12**	0.1331	0.3925	0.1256	0.0406
**Patient13**	0.6687	0.8629	1.6675	0.3573
**Patient14**	1.3953	0.646	1.0329	0.6052
**Patient15**	2.6321	1.6992	0.9797	0.6007
**Patient16**	0.0615	0.4441	0.0553	0.2211
**Patient17**	0.0674	0.0763	0.0763	0.0301
**Patient18**	0.1946	0.8631	0.6485	0.1989
**Patient19**	1.2079	1.1885	0.2629	0.2374
**Patient20**	2.021	1.8163	1.1856	0.5838
**Patient21**	0.3915	0.6838	0.4642	0.4927
**Patient22**	1.7514	0.9472	0.112	0.0259
**Patient23**	2.7133	0.1318	0.2942	0.0666
**Patient24**	2.223	0.3833	1.1384	0.5263
**Patient25**	0.9302	0.7154	0.8771	0.48
**Patient26**	0.0134	0.5752	0.0419	0.0316
**Patient27**	1.6408	1.5948	1.0612	0.7979
**Patient28**	0.6528	0.6274	0.1055	1.1741
**Patient29**	0.8309	0.9402	0.9843	1.2334
**Patient30**	0.0828	0.0539	0.0663	0.0227
**Patient31**	0.8513	0.0474	0.5182	0.0091
**Patient32**	0.0092	0.1414	0.0248	0.0203
**Patient33**	1.222	0.0974	1.7817	0.0579
**Patient34**	1.0433	1.9676	0.7693	0.6068
**Patient35**	0.247	0.9842	0.3182	0.0291
**Patient36**	0.1601	0.7266	0.0941	0.0733
**Patient37**	2.9111	0.3449	0.3988	0.3476
**Patient38**	0.0891	0.0583	0.0301	0.0111
**Patient39**	4.3328	0.1484	0.2274	0.1635
**Patient40**	3.5075	0.1324	0.0245	0.0486

**Patient41**	8.9479	1.5382	3.0217	0.9244
**Patient42**	1.8199	0.1501	0.0666	0.0541
**Patient43**	0.0964	0.0417	0.0275	0.041
**Patient44**	10.7701	0.1794	2.9778	0.1885
**Patient45**	1.3001	0.6362	0.6196	0.0196
**Patient46**	0.5568	1.0107	0.5072	0.7091
**Patient47**	1.5233	0.7623	0.0781	0.6767
**Patient48**	2.9192	0.5027	3.0001	0.4926
**Patient49**	7.943	1.3261	0.107	0.1197
**Patient50**	3.7136	0.101	0.0824	0.0451
**Patient51**	0.5136	0.0354	0.0139	0.0229
**Patient52**	1.6379	0.6386	0.0624	0.2814
**Patient53**	1.6725	0.6802	1.025	0.5789
**Patient54**	0.4593	0.7663	0.2726	0.2157
**Patient55**	1.9776	1.0877	1.9224	0.7161
**Patient56**	3.029	1.2754	1.2302	1.3519
**Patient57**	4.2972	0.1825	0.1209	0.2077
**Patient58**	2.7879	1.128	0.2522	0.9556
**Patient59**	2.477	1.2204	0.8038	0.5341
**Patient60**	1.1626	0.1873	0.1441	0.1017
**Patient61**	4.7457	0.3593	5.9216	0.4942
**Patient62**	0.0888	1.1369	0.175	1.0093
**Patient63**	3.3909	0.7168	1.8737	0.6753
**Patient64**	0.7278	0.456	0.6503	0.2921
**Patient65**	3.6419	1.1073	2.0594	0.5112
**Patient66**	3.8453	1.229	3.6988	1.1679
**Patient67**	1.401	0.9609	0.3735	0.3429
**Patient68**	0.2383	0.0604	0.0667	0.057
**Patient69**	6.3307	0.1623	1.4695	0.1182
**Patient70**	3.5791	0.9575	0.8799	0.4298
**Patient71**	4.9398	0.6673	3.5636	0.5014
**Patient72**	1.8227	1.2946	0.6457	0.9127
**Patient73**	5.7599	0.1081	0.0762	0.0954
**Patient74**	1.1349	0.4632	0.7727	0.4683
**Patient75**	0.0741	0.1051	0.1271	0.1206
**Patient76**	7.2763	0.3065	0.0427	0.1015
**Patient77**	0.7775	0.16	0.0163	0.024
**Patient78**	3.0112	0.9331	1.8804	1.1438
**Patient79**	8.8329	1.2211	1.0192	0.5536
**Patient80**	2.5331	0.613	0.909	0.5916
**Patient81**	0.2354	0.3641	0.2227	0.1352
**Patient82**	2.2886	0.5901	0.1026	0.0972
**Patient83**	1.0349	1.0459	0.517	0.7846
**Patient84**	9.1641	0.0696	0.0469	0.0373

**Patient85**	1.7749	0.7071	0.5082	0.5723
**Patient86**	0.0487	0.0761	0.0952	0.032
**Patient87**	0.2364	0.1724	0.1803	0.0547
**Patient88**	0.4267	1.8225	2.6342	0.5005
**Patient89**	2.5151	1.914	0.3362	0.2828
**Patient90**	1.2311	0.8607	0.5343	0.7652
**Patient91**	19.8914	2.1416	5.7191	1.0986
**Patient92**	1.3194	0.5599	0.9099	0.7524
**Patient93**	0.3965	2.2952	1.6997	0.6381
**Patient94**	2.5292	1.8019	1.735	1.5187
**Patient95**	1.5039	0.0563	0.619	0.0097
**Patient96**	1.6662	0.0703	0.0222	0.029
**Patient97**	7.8834	3.7457	2.5245	0.0314
**Patient98**	0.043	0.2199	0.0239	0.0135
**Patient99**	0.0602	0.0663	0.0363	0.0105
**Patient100**	0.0792	0.1155	0.0645	0.0308
**Patient101**	1.0608	0.3499	0.3253	0.0576
**Patient102**	0.0111	0.023	0.016	0.0057
**Patient103**	5.0088	3.8364	0.1415	1.0696
**Patient104**	2.8429	0.3234	0.7362	0.3454
**Patient105**	0.602	0.5201	0.5798	0.6035
**Patient106**	1.6517	0.6862	0.7521	0.4515
**Patient107**	0.7926	1.2499	1.8746	0.1785
**Patient108**	4.5186	0.0901	0.0209	0.0304
**Patient109**	0.8927	0.8919	0.8277	0.7425
**Patient110**	1.5705	1.3677	0.626	0.7442
**Patient111**	0.7304	0.7564	0.4828	0.9134
**Patient112**	2.0702	0.781	0.8651	0.8276
**Patient113**	1.2093	1.0782	1.0998	0.7589
**Patient114**	5.5238	0.1418	0.0447	0.0308
**Patient115**	6.1799	2.9776	4.3813	1.1169
**Patient116**	0.7552	0.9322	0.8107	0.6934
**Patient117**	0.9604	0.6514	1.142	0.7548
**Patient118**	2.4642	0.3975	0.6116	0.3857
**Patient119**	2.4711	0.7668	0.0667	0.3415
**Patient120**	0.1434	0.181	0.6209	0.0592
**Patient121**	2.5753	0.8419	1.0003	0.498
**Patient122**	4.2256	3.8528	1.0073	1.7538
**Patient123**	1.9527	0.4455	0.0888	0.6037
**Patient124**	1.7715	0.6291	0.7631	0.4641
**Patient125**	0.4327	1.4518	0.4895	0.4951
**Patient126**	7.7413	0.8003	0.3873	0.9186
**Patient127**	0.3488	0.8148	0.229	0.6516
**Patient128**	2.8906	0.5223	0.3577	0.4272

Supplementary Table 3. Diagnostic test of responses of RCO to combined chemoradiation for predicting the clinical outcomes of LARC patients in the discovery cohort, Related to Figure 2.

**Organoids**	**Clinical Outcomes**		
	good	poor	Total
sensitive	36	5	41
resistant	1	38	39
Total	37	43	80

Accuracy(%)=(36+38)/(41+49)=92.50%

Sensitivity(%)=38/(38+5)=88.37%

Specifity(%)=36/((36+1)=97.30%

Supplementary Table 4. A chi-square test of independence to examine the relation between RCO responses to cominbed chemoradiation and single treatments to predict the clnical outcomes of LARC patientsin the discovery cohort, Related to Figure2.

**Combined chemoradiation**	**Single treatments**		
	sensitive	resistant	Total
sensitive	38	3	41
resistant	3	36	39
Total	41	39	80

Supplementary Table 5. Diagnostic test of responses of RCO to combined chemoradiation for predicting the clinical outcomes of LARC patients in the validation cohort, Related to Figure 2.

**Organoids**	**Clinical Outcomes**		
	good	poor	Total
sensitive	19	1	20
resistant	2	26	28
Total	21	27	48

Accuracy(%)=(19+26)/(20+28)=93.75%

Sensitivity(%)=26/(26+1)=96.30%

Specifity(%)=19/((2+19)=90.48%

Supplementary Table 6. Diagnostic test of responses of RCO to single treatments for predicting the clinical outcomes of LARC patients in the validation cohort, Related to Figure 2.

**Organoids**	**Clinical Outcomes**		
	good	poor	Total
sensitive	17	4	21
resistant	4	23	27
Total	21	27	48

Accuracy(%)=(17+23)/(21+27)=83.33%

Sensitivity(%)=23/(23+4)=85.19%

Specifity(%)=17/((4+17)=80.95%

Supplementary Table 7. A chi-square test of independence to examine the relation between RCO responses to cominbed treatments and single treatments to predict the clnical outcomes of LARC patients in the validation cohort, Related to Figure 2.

**Combined chemoradiation**	**Single treatments**		
	Matched	Not matched	Total
Matched	18	2	20
Not matched	3	25	28
Total	21	27	48

Supplementary Table 8. Four modes of organoids responses to combined chemoradiation and tumor regression of corresponding clinical patients, Related to Figure 3

**Patients number**	**Four categories**	**Clinical response**
**Patient1**	rapid	0
**Patient2**	recovery	3
**Patient3**	resistant	2
**Patient4**	rapid	1
**Patient5**	resistant	2
**Patient6**	moderate	1
**Patient7**	recovery	3
**Patient8**	rapid	1
**Patient9**	resistant	3
**Patient10**	resistant	2
**Patient11**	resistant	2
**Patient12**	rapid	0
**Patient13**	resistant	2
**Patient14**	resistant	2
**Patient15**	resistant	2
**Patient16**	moderate	ccr
**Patient17**	rapid	0

**Patient18**	rapid	1
**Patient19**	moderate	1
**Patient20**	resistant	3
**Patient21**	resistant	3
**Patient22**	moderate	0
**Patient23**	rapid	1
**Patient24**	resistant	0
**Patient25**	resistant	2
**Patient26**	rapid	0
**Patient27**	resistant	2
**Patient28**	resistant	2
**Patient29**	resistant	2
**Patient30**	rapid	0
**Patient31**	rapid	1
**Patient32**	rapid	0
**Patient33**	rapid	0
**Patient34**	resistant	2
**Patient35**	rapid	0
**Patient36**	rapid	0
**Patient37**	recovery	2
**Patient38**	rapid	ccr
**Patient39**	rapid	ccr
**Patient40**	rapid	ccr
**Patient41**	resistant	2
**Patient42**	moderate	ccr
**Patient43**	moderate	0
**Patient44**	moderate	0
**Patient45**	rapid	1
**Patient46**	resistant	2
**Patient47**	resistant	3
**Patient48**	resistant	3
**Patient49**	moderate	0
**Patient50**	rapid	3
**Patient51**	rapid	0
**Patient52**	moderate	0
**Patient53**	resistant	2
**Patient54**	rapid	1
**Patient55**	resistant	2
**Patient56**	resistant	2
**Patient57**	rapid	0
**Patient58**	resistant	2
**Patient59**	recovery	3
**Patient60**	rapid	0
**Patient61**	resistant	2

**Patient62**	resistant	2
**Patient63**	resistant	2
**Patient64**	rapid	1
**Patient65**	resistant	2
**Patient66**	resistant	2
**Patient67**	resistant	2
**Patient68**	rapid	0
**Patient69**	moderate	1
**Patient70**	recovery	2
**Patient71**	recovery	2
**Patient72**	resistant	3
**Patient73**	rapid	3
**Patient74**	recovery	2
**Patient75**	rapid	1
**Patient76**	rapid	2
**Patient77**	rapid	1
**Patient78**	resistant	2
**Patient79**	resistant	2
**Patient80**	resistant	3
**Patient81**	rapid	0
**Patient82**	rapid	0
**Patient83**	resistant	2
**Patient84**	rapid	0
**Patient85**	resistant	2
**Patient86**	rapid	ccr
**Patient87**	rapid	ccr
**Patient88**	recovery	2
**Patient89**	moderate	ccr
**Patient96**	rapid	0
**Patient103**	resistant	2
**Patient107**	moderate	ccr
**Patient125**	resistant	3
**Patient126**	resistant	2
**Patient127**	resistant	2
**Patient128**	resistant	2

Supplementary Table 9. A chi-square test of independence to examine the relation among the RCO responses (sensitive or resistant) to irradiation, 5-Fu and CPT-11 in 128 organoid lines, Related to Figure 2.

**Treatments**	**Organoids response**		
	Sensitive	Resistant	Total
Radiation	24	104	128
5-Fu	39	89	128

CPT-11	48	66	114

Supplementary Table 10. A fisher's exact test of independence to examine the relation among the RCO responses (only sensitive or not) to irradiation, 5-Fu and CPT-11 in 128 organoid lines, Related to Figure 2.

**Treatments**	**Organoids response**		
	Sensitive (Only)	Others	Total
Radiation	2	126	128
5-Fu	8	120	128
CPT-11	14	100	114

Supplementary Table 11. Organoids size change ratios on day24 to day0 after treatment with single or combined regimens from 10 cases, Related to Figure 2 and Figure 4.

	Irradiation	5Fu	CPT11	Oxaliplatin	5FU + CPT11	5FU + Oxaliplatin	5FU + CPT11 + Oxaliplatin
**Patient-1**	0.227	1.446	0.182	5.162	0.183	1.041	0.086
**Patient-2**	0.699	3.929	0.267	2.057	0.481	1.281	0.385
**Patient-3**	1.393	2.472	1.463	5.415	1.901	1.911	1.256
**Patient-4**	0.569	0.983	0.495	0.805	0.775	0.806	0.776
**Patient-5**	0.173	0.422	1.160	0.068	0.103	0.112	0.099
**Patient-6**	0.892	0.516	0.123	1.219	0.209	0.528	0.339
**Patient-7**	0.553	2.462	0.272	9.933	0.937	2.444	0.887
**Patient-8**	1.194	0.683	2.424	1.268	0.889	0.499	0.799
**Patient-9**	0.238	0.541	0.136	0.313	0.339	0.452	0.142
**Patient-10**	1.107	0.950	0.982	1.061	1.039	0.798	0.675

Supplementary Table 12. Specific details of the organoid culture conditions, Related to STAR METHOD.

Regent name	Company	Cat No.	Stock	Solvent	Final
Advanced DMEM/F12	Gibco	12634-010	---	1×	1×
R-spondin 1	Sino	11083-	50μg/ mL	0.1%BSA/P	500ng/mL
Noggin	Sino	50688-	10μg/ mL	0.1%BSA/P	100ng/mL
R-spondin 1	EnaMab	ERSP1000	50μg/ mL	0.1%BSA/P	500ng/mL
Noggin	EnaMab	ENGG0100	10μg/ mL	0.1%BSA/P	100ng/mL
Wnt3a	EnaMab	EWNT0100	10μg/ mL	0.1%BSA/P	100ng/mL
EGF	Sino	50482-	500μg/ mL	0.1%BSA/P	50ng/mL
HEPES	Gibco	15630080	100×	---	1×
Glutamax	Gibco	35050061	100×	---	1×
Normocin	InvivoGen	ant-nr-1	500×	---	1×
Gentamicin/amphotericin	Gibco	R01510	500×	---	1×
N2	Invitrogen	17502-048	50×	---	1×
B27	Invitrogen	17504-044	100×	---	1×
n-Acetylcysteine	Sigma-	A9165	500mM	ddH2O	1mM
Niacinamide	Sigma-	N0636	1M	ddH2O	10mM
A-83-01	Tocris	2939	5mM	DMSO	500nM
SB202190	Sigma-	S7067	30M	DMSO	3μM
Gastrin	Sigma-	G9145	100uM	0.1%BSA/P	10nM
Prostaglandin E2	Sigma-	P6532	100uM	DMSO	10nM
